# Isotherm, kinetics, and optimization modeling of Cr(VI) ions and methylene blue dye adsorption from water by an aminobiochar hydrogel

**DOI:** 10.1038/s41598-026-49810-7

**Published:** 2026-05-04

**Authors:** Omnia Fakih Mousa, Murat Yılmaz, Mohamed A. El-Nemr, Mohamed A. Hassaan, Amel M. Ismail, Ahmed El Nemr

**Affiliations:** 1 Department of Chemistry, Faculty of Science, Alexandria University, Alexandria, Egypt; 2 Department of Chemistry and Chemical Processing Technologies, Bahçe Vocational School, Osmaniye Korkut Ata University, Osmaniye, 80000 Turkey; 3 Department of Chemical Engineering, Faculty of Engineering, Minia University, Minia, 61519 Egypt; 4 The Higher Canal Institute of Engineering and Technology, Al Salam 1 - Abu Bakr Al Siddiq Street, Suez, Egypt; 5 Environment Division, National Institute of Oceanography and Fisheries (NIOF), Kayet Bey, Elanfoushy, Alexandria, Egypt

**Keywords:** Aminobiochar hydrogel, Orange peel biochar, Adsorption, Chromium (VI), Methylene blue, Response surface methodology, Artificial neural network, Chemistry, Environmental sciences, Materials science

## Abstract

This work presents a novel hydrogel produced from a newly manufactured aminobiochar with a high adsorption capacity for the removal of various pollutants from water. This study investigates the adsorption of Cr (VI) ions and methylene blue (MB) dye using a fabricated aminobiochar hydrogel (ABHG) synthesized from orange peels via microwave-assisted sulfuric acid activation, followed by oxidation, amination, and hydrogel formation. The ABHG exhibited a high surface area and diverse functional groups, confirmed by BET, SEM, FTIR, and TGA analyses. The FTIR analysis shows the presence of C–H, C≡C, C=C, C=O, and SO_3_ groups on the surface of the prepared hydrogel adsorbent. Under optimal conditions (initial concentration of 25 mg/L for both MB dye and Cr (VI) ions, contact time of 90 min for MB dye and 180 min for Cr (VI), pH 7.2 for MB dye and 1.04 for Cr (VI), and temperature 25 °C), maximum removal efficiency of both contaminants was achieved. Optimal adsorption experiments demonstrated maximum capacities of 476.19 mg/g for MB dye and 1250.00 mg/g for Cr (VI) ions based on the Langmuir isotherm model. Kinetic analysis showed MB dye adsorption followed a pseudo-second-order model, whereas Cr (VI) adsorption fitted a pseudo-first-order model. Using response surface methodology (RSM), the highest removal efficiencies were achieved with 0.958 g ABHG for 59.78 mg/L Cr (VI) and 1.91 g ABHG for 27.41 mg/L MB dye. Artificial neural network (ANN) modeling further validated these findings, confirming the potential of ABHG as an effective adsorbent for wastewater treatment.

**Supplementary Information:**

The online version contains supplementary material available at 10.1038/s41598-026-49810-7.

## Introduction

Water contamination is a pressing worldwide issue in the twenty-first century, caused by industrialization, urban growth, and irresponsible use of natural resources, that adversely affects the ecological environment and human well-being^1^. Chromium (Cr) is a toxic heavy metal element commonly present in industrial effluent, typically in the Cr (III) and Cr (VI) valence states^2^. Cr (VI) is believed to be 100 to 1000 times more toxic than Cr (III), and even a minute amount can result in devastating health consequences, including mutagenicity and carcinogenicity^3,4^. Synthetic dyes are notoriously polluting because of their widespread use across sectors such as paper, cosmetics, textiles, printing, tanneries, and food^5–7^. Synthetic dyes are generally classified into three categories based on their dissociation behavior in aqueous solutions: anionic (acid, direct, and reactive dyes), cationic (basic dyes), and nonionic (disperse dyes)^8^. Methylene blue (MB) is a cationic dye commonly used for dyeing goods, including wood, silk, and cotton^9,10^. The removal of MB dye and Cr(VI) ions from water is critically important due to their distinct structural properties and widespread use in various industrial applications. Currently, several techniques have been devised to eliminate dyes and heavy metals, including membrane filtration^11^, advanced oxidation^12^, adsorption^13,14^, photocatalytic degradation^15,16^, and biological treatment^17,18^. The adsorption method stands out among these approaches because of its high removal efficiency, high availability, lack of by-products, straightforward operation, and low cost. Adsorption has been extensively employed for the removal of heavy metals and ammonia nitrogen from wastewater^19–21^.

Activated carbon is the primary adsorbent in several processes for the treatment of dyes and heavy metals^22,23^. However, the use of activated carbon is constrained by its high cost and the challenges of its regeneration^24,25^. Utilizing inexpensive raw materials, such as palm petiole^26^, sawdust^27^, spinach waste^28^, date palm kernel^29^, rice husk^30^, spent coffee grounds^31^, cocoa pods^32^, red algae^33^, watermelon peel^34^, mandarin peel^35^, pea pods^36^, chitosan^37^, cotton-straw^38^, and soybean stover^39^ have been explored as a potential remedy for this problem. These carbons were employed as cost-effective adsorbents to eliminate metal ions, dyes, and drug contaminants from effluents.

Recently, hydrogels have attracted considerable attention in environmental remediation due to their three-dimensional crosslinked polymeric structure, which enables them to absorb large amounts of water and swell without dissolving^40^. Their tunable surface chemistry, biocompatibility, and high adsorption capacities make them ideal candidates for removing pollutants from aqueous environments^41^. In particular, modified biochar-based hydrogels offer synergistic advantages, combining the porosity and surface activity of biochar with the network-forming capability and flexibility of hydrogels^42^. Such materials can provide enhanced interaction sites for heavy metal ions and organic dyes via electrostatic attraction, hydrogen bonding, ion exchange, and π–π interactions^42^. He et al. studied the removal of Cr (VI) ions from aqueous media by an alginate gel bead composite adsorbent containing polyethylenimine (PEI) and Eichhornia crassipes (EC) biochar as surface modifier, known as EC-alg/PEI-3, and reported the maximum adsorption capacities of EC-alg/PEI-3 as 714.3 mg g^−1^ at 10 °C and 769.2 mg g^−1^ at 25 °C^43^. Du et al. reported that a novel composite hydrogel (AM/CMC/B) synthesized from peanut shell biochar effectively adsorbed the heavy metal Cd in water, reducing its toxicity to tobacco seedlings. In this study, the hydrogel prepared via hydrothermal polymerization using acrylamide (AM), carboxymethyl cellulose (CMC), and peanut shell biochar exhibited a maximum adsorption capacity of 164.83 mg g^−1^ for Cd^2+^ and was reported to fit the pseudo-second-order kinetic model^44^. Consequently, integrating functionalized biochar into hydrogel matrices is a promising strategy for developing sustainable, efficient adsorbents for wastewater treatment.

Through pyrolysis, activated carbon can be generated from this biomass source using either chemical activation (e.g., H_3_PO_4_ or ZnCl_2_) or physical activation (e.g., CO_2_, H_2_O, or O_2_), resulting in materials with high surface area^45^. Biochars possess more functional groups than activated carbons but have lower pore volumes and surface areas^46–48^. The specific quantity and nature of functional groups in biochar will directly affect its efficacy in wastewater removal. The chemical modification of the biochar surface enables achieving this objective. Alternative methods to enhance the adsorption capability of biochars include nanoscale production, oxidation, surface activation, and metal impregnation^49^. The adsorption capacity of biochar can be enhanced by introducing amino groups onto its surface^47^. Chemically modifying biochar with different acids (H_3_PO_4_, H_2_SO_4_, or HNO_3_), bases (NaClO, or KOH), or oxidizing agents (NH_3_H_2_O, (NH_4_)_2_ S_2_O_8_, H_2_O_2_, KMnO_4_, or NaClO) can increase the number of functional groups present^46,50,51^.

This study chose orange peels as the primary source for synthesizing highly porous biochar^52^. Orange peels are readily available and cost-effective materials that are frequently wasted by operators of fresh juice outlets and fruit stands^53^. In 2012, the Food and Agriculture Organization (FAO) projected that worldwide orange production would exceed 68 million tonnes, with Egypt contributing more than 2.7 million to that figure. The composition of orange peel percentage by dry weight is 2.3% fat, 12.6% hemicellulose, 7.8% protein, 51.1% pectin, 11.1% cellulose, 4.5% starch, 1.0% lignin, and 4.2% ash^53^.

Despite the extensive use of agricultural residues for adsorbent preparation, no previous study has reported microwave-assisted H_2_SO_4_ activation of orange peel-derived biochar followed by sequential oxidation and amination to fabricate aminobiochar hydrogel (ABHG). This unique synthetic route produced a highly porous, nitrogen- and oxygen-functionalized carbon structure, as confirmed by Brunauer–Emmett–Teller (BET), Scanning Electron Microscopy (SEM), Fourier-transform infrared spectroscopy (FTIR), and Thermogravimetric analysis (TGA). Microwave irradiation significantly reduced activation time (3 min), while oxidation and amination introduced amine, hydroxyl, and carboxyl functionalities that enhanced affinity toward both cationic and anionic pollutants.

This study is further distinguished by the simultaneous removal of methylene blue (MB) and Cr (VI)—model contaminants frequently co-present in textile, electroplating, and tanning effluents. Evaluating both pollutants demonstrates the adsorbent’s versatility and real-world applicability. Kinetic and isotherm analyses revealed different interaction mechanisms for MB and Cr(VI), while Response Surface Methodology (RSM) and Artificial Neural Networks (ANN) modeling provided reliable multivariate optimization and predictive performance.

This work introduces a novel activation-functionalization pathway for orange-peel-derived biochar, enabling rapid microwave-assisted synthesis of amino-functionalized biochar hydrogel (ABHG). Notably, ABHG exhibits exceptional adsorption capacity for the simultaneous removal of hexavalent chromium (Cr (VI)) and methylene blue (MB) dye from wastewater. Integrating an artificial neural network (ANN) and response surface methodology (RSM) modelling optimizes the process with high precision. These findings underscore the potential of ABHG as a cost-effective, multifunctional adsorbent for sustainable wastewater treatment.

## Materials and methods

### Materials and equipment

Orange peel (OP) was procured from local retail establishments in Alexandria, Egypt. The following chemicals were obtained from ADWIC, El Nasr pharmaceutical chemical company, Egypt: Hydrochloric acid (HCl) with a molecular weight (M.W.) of 36.46 g/mol, as determined through an acidimetric assay (37%), sodium hydroxide (NaOH) with an M.W. of 40 g/mol, as determined by a minimum assay threshold of 96%, potassium dichromate powdered (K_2_Cr_2_O_7_, M.W. = 294.18 g/mol, minimum assay 99.8%), and glutaraldehyde solution (C_5_H_8_O_2_, Assay about: 50%) of Aldrich, USA. Nitric acid (HNO_3_, M.W. = 63.01 g/mol, Assay Approximate: 65.0%) and methanol (CH_3_OH, M.W. = 32.04 g/mol, Assay Approximate: 99.99%) were obtained from Fisher Scientific UK company, located in Loughborough, UK. The following chemicals were obtained from Sigma Aldrich Company, USA: Polyvinylpyrrolidone (PVP, K15) BioChemica, M.W. = approx. 10,000 (g/mol), Assay (N) 12.0–12.8%, and Polyvinyl alcohol (PVA (–C_2_H_4_O)_n_, M.W. = 30.000–70.000, hydrolysis (87–90%). Sulfuric acid (H_2_SO_4_, M.W. = 98.07 g/mol, assay content of about 98.0%) was acquired from SD Fine-Chem Limited (SD FCL) in Mumbai, India. The phosphorus (V) oxide (P_2_O_5_), with a mass of 141.94 g and an acidimetric assay of at least 97.0%, was obtained from Research-Lab Fine Chem Industries in Mumbai, India. A solution of ammonia (NH_4_OH) with an assay concentration of around 25% was procured from Pharaohs Company for commerce and import operations in Egypt. The compound 1,5-Diphenylcarbazide (C6H5NHNHCONHNHC6H5, M.W. = 242.28 g/mol) was acquired from BDH Chemicals Ltd, located in Poole, England. The compound methylene blue (basic blue 9; C.I.52015), with the chemical formula C_16_H_18_ClN_3_S (M.W. = 319.85 g/mol), was acquired from Honeywell Riedel-de Haën AG, located in Seelze, HANNOVER, Germany. In all experimental studies, double-distilled water was used, and each experiment was conducted in triplicate. Only the average values were used for all calculations reported in this manuscript.

The pollutant concentration was determined using a UV–visible spectrophotometer (Analytic Jena, model SPEKOL1300, UK) equipped with 1 cm glass cells. This study utilized a SANYO Microwave Oven (Model No. EM-D975W-Max; input 1400 W), Egypt; a JENCO pH meter (model 6173); a Heating magnetic stirrer (VELP-Code F20500010), Italy; and a JS shaker (model JSOS-500), Korea, for the experiments. Fourier Transform Infrared (FTIR) spectroscopy was employed to evaluate the adsorbent surface, specifically using the platinum attenuated total reflection (ATR) model V-100 VERTEX70 (Germany). The methodology above facilitated the identification of functional groups on the surface within the wave-number spectrum spanning 400–4000 cm^−1^. The Scanning Electron Microscope (SEM) instrument (LEO 1450 VP, USA) was used to analyze the elemental composition and surface morphology of activated carbon samples. The SEM was integrated with an Energy Dispersive X-ray Spectrometer (EDX) for elemental analysis.

### Preparation of the raw material

The Orange peel (OP) sample was rinsed with tap water and washed multiple times, followed by drying in an oven at 50 °C until completely desiccated. Once the peels had dried, they were crushed and filtered through a 100 µm sieve. The 100 μm sieve size was selected to ensure a uniform particle size distribution, thereby enhancing surface area exposure during activation and subsequent reactions. This size range is commonly used in biochar and adsorbent studies to improve process consistency and adsorption efficiency^54,55^.

### Preparation of biochar

Biochar was synthesized by introducing 3 g of OP sample into a quartz reactor and combining it with 10 mL of 50% H_2_SO_4_ solution. The mixture was then placed in a domestic Microwave oven (1400 W) and activated for 3 min. Upon completion of the activation period, the carbonized biochar samples were removed from the microwave and cooled. Subsequently, the specimens underwent multiple washes with distilled water until a pH range of 6–7 was achieved. The washed biochar was dried in the oven at 50 °C for 24 h to yield 1.56 g (52% of OP), thereafter crushed and sieved to a particle size < 100 µm, and preserved in a glass bottle for further use (Fig. S1). The quantification of biochar yield was determined by employing the subsequent Eq. (1):

1 $$Y\left( \% \right) = \frac{{W_{2} }}{{W_{1} }} \times 100$$

where *W*_*1*_ (g) denotes the mass of the raw materials before activation, and *W*_*2*_ (g) denotes the mass of the generated biochar post-activation^56^.

### Oxidation of biochar and modification with ammonia solution

The oxidation process involved adding 10 g of biochar to a solution containing 40 mL of concentrated HNO_3_ in 100 mL of cold water. The mixture was then agitated in an ice bath for 10 min. After 10 min, 20 g of P_2_O_5_ was introduced and agitated for 1 h at 5 °C. After oxidation treatment, the supernatant was separated. The remaining solid was subsequently rinsed with distilled water, oven-dried at 50 °C for 48 h to yield 11.25 g, and finally crushed before being stored in glass bottles. Afterward, a 250 mL necked flask was combined with approximately 5 g of oxidized biochar and 100 mL of 25% NH_4_OH. The flask was equipped with a condenser and a heated magnetic stirrer. The mixture was then refluxed overnight. Following reflux, the supernatant was transferred to a beaker and evaporated at 100 °C for 4 h, after which it was dried in an oven at 50 °C for 72 h to yield 6.75 g, then ground to a fine powder and stored in glass bottles^36,47^.

### Preparation of aminobiochar hydrogel

A 5 g sample of polyvinyl alcohol (PVA) with a molecular weight between 30,000 and 70,000 was evenly distributed in 50 mL of water using a heated magnetic stirrer set at 90 °C. The dispersion process was continued until the dispersion was achieved. Subsequently, ammonia-modified biochar (100 mg) was added to the PVA dispersion at room temperature. To achieve cross-linking between PVP and aminobiochar, add 1.5 mL Glutaraldehyde as a cross-linker and 2 drops HCl as a catalyst. Stirring was continued for 15 min, after which the mixture was placed in an oven overnight at 50 °C to facilitate the reaction by releasing water and producing a gel^40–42^. The gel was rinsed with water to produce aminobiochar hydrogel, which was labeled ABHG (Fig. S1).

### Characterization

The surface morphology of the ABHG sample was analyzed using a QUANTA 250 scanning electron microscope (SEM). Fourier transform infrared (FTIR) spectroscopy was employed to ascertain the surface functional groups in the ABHG structure. The V-100, a platinum ATR structure connected to a Bruker VERTEX70 model, Germany, was employed to determine the IR-observable functional groups present on the carbon surface within the wave number range of 400–4000 cm^−1^. The pollutant concentration was measured using a UV–visible spectrophotometer (Analytic Jena, model SPEKOL1300) equipped with 10 mm glass cells. The experiment was conducted with a JENCO pH meter (model 6173) and a JS shaker (model JSOS-500). Thermal assessments were performed utilizing the SDT650 Simultaneous Thermal Analyzer. The device has a temperature range spanning from ambient conditions up to 1000 °C, with a ramp rate of 5 °C per minute. The nitrogen gas adsorption–desorption isotherm on activated carbon was obtained at the boiling point of N_2_ gas. The activated carbon’s BET surface area (*S*_*BET*_ = 75.16 m^2^/g) was determined by measuring N_2_ adsorption at 77 K utilizing the BELSORP—Mini II analyzer device manufactured by BEL Japan, Inc. An isotherm BET analysis was conducted to determine the surface area (*S*_*BET*_) in m^2^ g^−1^, monolayer volume (*V*_*m*_ = 17.27 cm^3^ (STP) g^−1^), energy constant (*C* = 727.13), total pore volume in *p*/*p*_0_ (*V*_*T*_ = 0.0515 cm^3^ g^−1^), and mean pore diameter (2.741 nm) (Fig. S2). The average pore radius was determined by applying Eq. (2):

2 $$r\left( {nm} \right) = \frac{{2V_{T} \left( {{\mathrm{mLg}}^{ - 1} } \right)}}{{a_{s,BET} \left( {{\mathrm{m}}^{2} {\mathrm{g}}^{ - 1} } \right)}} \times 1000$$

The Barrett-Joyner-Halenda (BJH) technique was applied to calculate the mesopore volume (*V*_*me*_ = 0.02676 cm^3^/g), and mesopore surface area (*S*_*me*_ = 19.584 m^2^/g) of ABHG. These measurements were obtained utilizing the BELSORP analysis software. The pore size distribution was determined from the desorption isotherm using the BJH method (Fig. S2)^46,47^. The TGA and DTA diagram of ABHG is shown in Fig. S3.

### MB dye and Cr (VI) ions adsorption experiments

A number of batch adsorption experiments were performed using Erlenmeyer flasks. The flasks were filled with methylene blue (MB) dye solutions at 50 mL, with an initial concentration of 50 mg/L. The experiment involved adding equal masses of activated carbon hydrogel containing 0.025 g of ABHG to MB dye solutions. The cube of the modified AC hydrogel was then cut, and each sample was placed on a shaker and shaken at 200 rpm at room temperature for 90 min to achieve equilibrium. A UV–visible spectrophotometer set to 665 nm was used to measure the concentration of MB in solution before and after adsorption. This procedure is the same as in the Cr (VI) ion adsorption experiments, with minor differences. The flasks were filled with solutions of Cr (VI) ions (100 mL) with a beginning concentration of 75 mg/L of ABHG. After cutting the cube of the modified biochar hydrogel, equal masses of ABHG were added to the Cr (VI) ions solution. Each sample was agitated on a shaker at 200 rpm and room temperature for 180 min to ensure equilibrium formation. Concentrations of the ion Cr (VI) in the solution were measured with a UV–visible spectrophotometer at a wavelength of *λ*_540_ nm, both before and after adsorption. Typically, we use the mean values from triblicate experiments in all calculations, such as kinetic and isotherm calculations and plots, where the standard deviation in this work is ± 1.65. The equilibrium adsorption capacity of the adsorbent, denoted as qe (mg/g), was determined using Eq. (3)^56^.

3 $${q}_{e}=\frac{\left({C}_{0}-{C}_{e}\right)V}{W}$$

where *C*_*0*_ denotes the beginning concentration of the MB dye or Cr (VI) ions (mg/L), *C*e represents the equilibrium concentration of the MB dye or Cr (VI) ions (mg/L), *V* indicates the volume of the solution (L), and *W* signifies the mass of the ABHG used (g).

All adsorption experiments were performed using synthetic aqueous solutions of Cr (VI) and methylene blue (MB) prepared in the laboratory. While synthetic systems provide controlled environments for mechanistic and performance studies, they may not fully represent the complex composition of real industrial wastewater. Future studies are planned to assess the performance of ABHG using actual effluent samples to validate its selectivity and practical applicability under realistic conditions.

#### Effect of pH

An examination of the influence of pH on the adsorption of MB dye or Cr (VI) ions^34–36,56^ was carried out. This was achieved by introducing 0.075 g of ABHG into 50 ml of MB dye solution with an initial concentration of 75 mg/L and an initial pH of 5–11. Similarly, 100 mL of Cr (VI) ion solutions with an initial concentration of 75 mg/L and an initial pH range of 1 to 9 were evaluated. The suspensions were stirred at 200 rpm for 90 min and 180 min, respectively, at ambient temperature. The pH of the solutions was adjusted using 0.1 M HCl or 0.1 M NaOH.

#### Effect of MB dye or Cr (VI) ions concentration

Various initial concentrations of MB dye (25–100 mg/L) or Cr (VI) ions (25–125 mg/L) were combined with 0.075 g of ABHG. Within these solutions, 50 mL and 100 mL were used to adsorb solutions of varying initial concentrations, respectively. The mixture was subsequently stirred at 200 rpm at ambient temperature for 90 and 180 min, respectively.

#### Effect of ABHG dosage

The impact of the ABHG dosage on the elimination of MB dye and Cr (VI) ions was investigated through the agitation of 50 and 100 mL of MB dye (25–100 mg/L) and Cr (VI) ions (25–125 mg/L) solutions, respectively, with varying dosages of adsorbents (0.025, 0.05, 0.075, and 0.1 g), for durations of 90 min and 180 min, respectively, at ambient thermal conditions.

#### Effect of time

The impact of contact duration on the elimination of MB dye and Cr (VI) ions was investigated for 90 and 180 min, respectively. Various amounts (0.025, 0.05, 0.075, and 0.1 g) of the adsorbent ABHG were added to 50 mL (25–100 mg/L) of MB dye and 100 mL (25–125 mg/L) of Cr (VI) ion solutions. The mixture was thereafter agitated with a shaker at 200 rpm. For each of the selected contact durations (15, 30, 45, 60, and 90 min) and (30, 45, 60, 120, and 180 min), the resulting mixtures were analyzed to determine the concentrations of residual pollutants^51,52,56^.

### Response surface methodology (RSM)

The optimization of parameters influencing the elimination of Cr (VI) and MB dye using the ABHG hydrogel adsorbent was investigated using response surface methodology (RSM). Design-Expert version 13.0.5.0 was used, and a D-Optimal design (DOD) was applied. The initial concentrations of Cr (VI) and MB, reaction duration, and adsorbent dose were selected for the RSM study. Tables 1 and 2 present the parameters under study and their respective levels, selected based on the results of the experimental trial. Cr (VI) and MB dye elimination percentage (%) were the responses under investigation. Twenty experiments were carried out using various combinations of factors. Tables 1 and 2 present the planned experiments.

**Table 1:** The range of parameters investigated for Cr (VI) ion removal utilized in the optimization process.

Factor	Name	Units	Minimum	Maximum	Mean	Std. Dev
A	Dose	g/L	0.25	1	0.6625	0.3272
C	Cr (VI) Conc	mg/L	25	125	80	43.38
B	Time	Min	30	180	109.50	63.29

**Table 2:** The range of parameters investigated for MB dye removal was utilized in the optimization process.

Factor	Name	Units	Minimum	Maximum	Mean	Std. Dev
A	Dose	g/L	0.5	2	1.15	0.6304
C	MB dye Conc	mg/L	25	100	62.50	31.93
B	Time	Min	15	90	54.75	30.11

### Artificial neural network (ANN) modeling

By biological human brain network simulation, ANN modeling predicts the correlations between input and output data. ANNs store and process large amounts of data using neurons. The feed-forward back-propagation neural network (BPNN) is one of the most widely used neural network architectures. A typical BPNN consists of an input layer (IL), representing the independent variables; one or more hidden layers (HLs); and an output layer (OL), representing the dependent variable. MATLAB R2015b version represented the elimination of Methylene blue and Cr (VI) via ABHG. The Levenberg–Marquardt (LM) training algorithm used 70% of the sample for training, 15% for validation, and 15% for testing. The correlation coefficient (*R*^2^) and mean squared error (MSE) are the main measures of ANN efficiency. The optimal network, characterized by the best-fit ANN model, is shown by the highest R^2^ and the lowest MSE. The optimal ANN model for MB removal by ABHG was a BPNN with a hidden layer of 9 neurons, while the best-fit model for Cr (VI) removal featured a hidden layer of 7 neurons. During the training phase, networks with 4 to 10 hidden-layer neurons were evaluated. The independent variables included the ABHG adsorbent dosage (g/L), contact time (min), and the initial concentration of MB or Cr (VI) (mg/L), while the dependent variable was the removal efficiency of MB or Cr (VI)^57–59^.

## Results and discussion

### Physico-chemical studies of ABHG

The present work involved the preparation of ABHG from agricultural waste, specifically orange peels, using microwave heating. The activation of this material was induced by 50% H_2_SO_4_ under microwave irradiation and subsequently changed using the following methods: The initial modification technique involves the oxidation of biochar using nitric acid and phosphorous pentaoxide, followed by a subsequent reaction of the oxidized activated carbon with ammonia solution under reflux. The present study uses PVA and Glutaraldehyde as crosslinkers to synthesize hydrogels from a modified biochar. Under the given circumstances, orange peels have the potential to form aminobiochar hydrogel.

Sulfuric acid, utilized as an activating agent, significantly influences modified adsorbents’ biochar yield and adsorption efficiency^60^. Following activation, the integration of sulfuric acid into the internal char matrix can impede tar formation and facilitate the entry of oxygen functionalities, as indicated by reaction^61^. Furthermore, the activation of H_2_SO_4_ can foster the formation of stable C-O complexes, thereby contributing to the observed internal porosities. Nevertheless, the biochar yield obtained by H_2_SO_4_ activation was comparatively limited. This can be attributed to the presence of water vapor resulting from the dehydration process of H_2_SO_4_, which has led to an increase in carbon burn-off^62^.

**Fig. 1:**
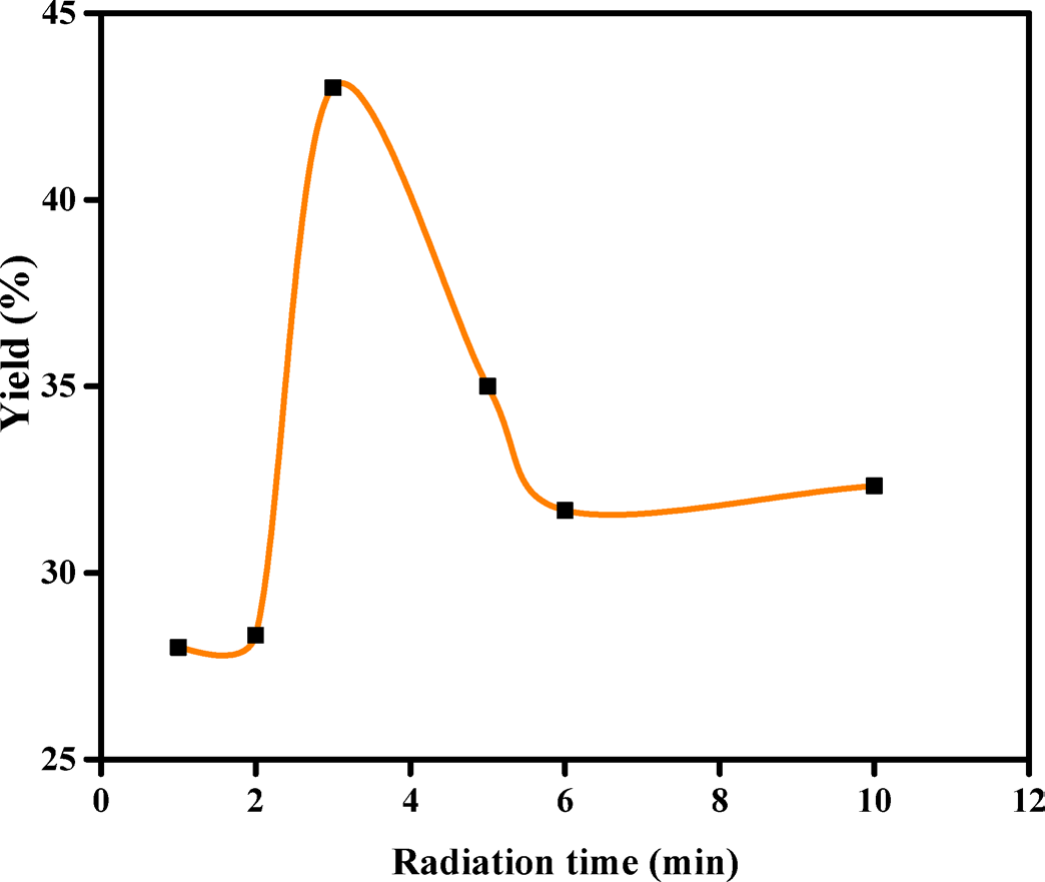
Effect of microwave irradiation time (preparation conditions: input microwave power = 1400 W) on the biochar yield in the presence of 50% H_2_SO_4_.

According to Fig. 1, activation of the orange peel raw material resulted in a relatively low biochar yield, ranging from 28 to 28.33% at radiation times of 1 and 2 min, respectively. It was observed that the orange peel was not completely burned at these time intervals. The maximum yield percentage of 43% was observed at a radiation time of 3 min. As the duration of radiation increased, the formation of water vapor from the dehydration of H_2_SO_4_ increased, leading to higher carbon burn-off and a decrease in the yield percentage. Therefore, the impact of H_2_SO_4_ as an activation agent on biochar yield was investigated at a microwave power of 1400 W and a radiation time of 3 min.

### Textural and morphological properties

**Fig. 2:**
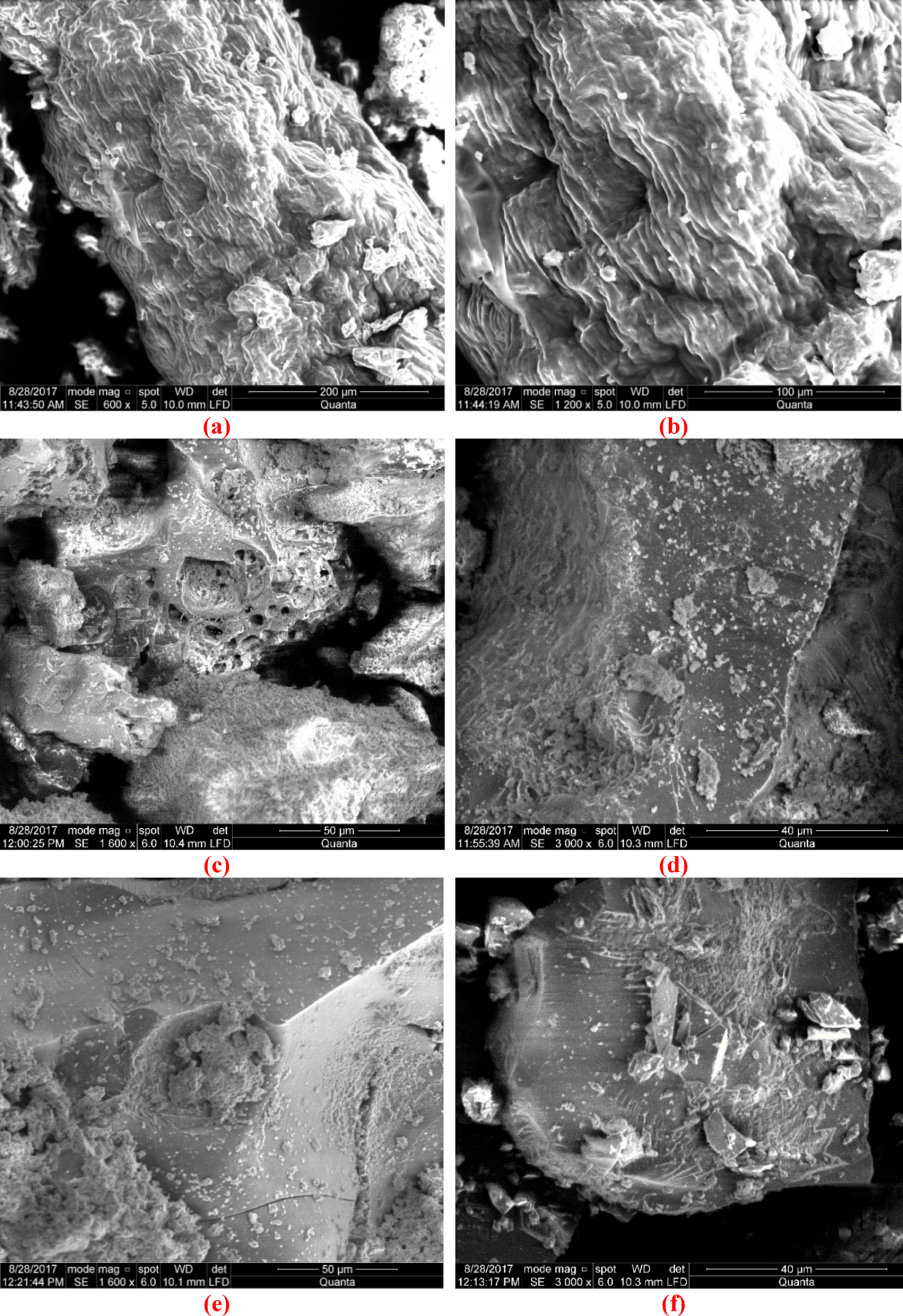
SEM Images of (**a**) Orange peel (600×, 200 µm), (**b**) biochar prepared by microwave-induced H_2_SO_4_ activation (1200×, 100 µm), (**c**) oxidized biochar prepared by oxidation with nitric acid and phosphorous pentaoxide (1600×, 50 µm), (**d**) ammonia biochar (3000×, 40 µm), (**e**) ABHG (1600× magnification) (1600×, 50 µm), (**f**) ABHG (3000× magnification) (3000×, 40 µm).

This study used SEM imaging to examine the surface morphology of the samples. The study involved the examination of SEM micrographs (Fig. 2) of orange peels (OP), microwave-activated biochar, H_2_SO_4_-activated biochar, and ammonia- and ABHG-modified biochar.

### Surface functional groups

**Fig. 3:**
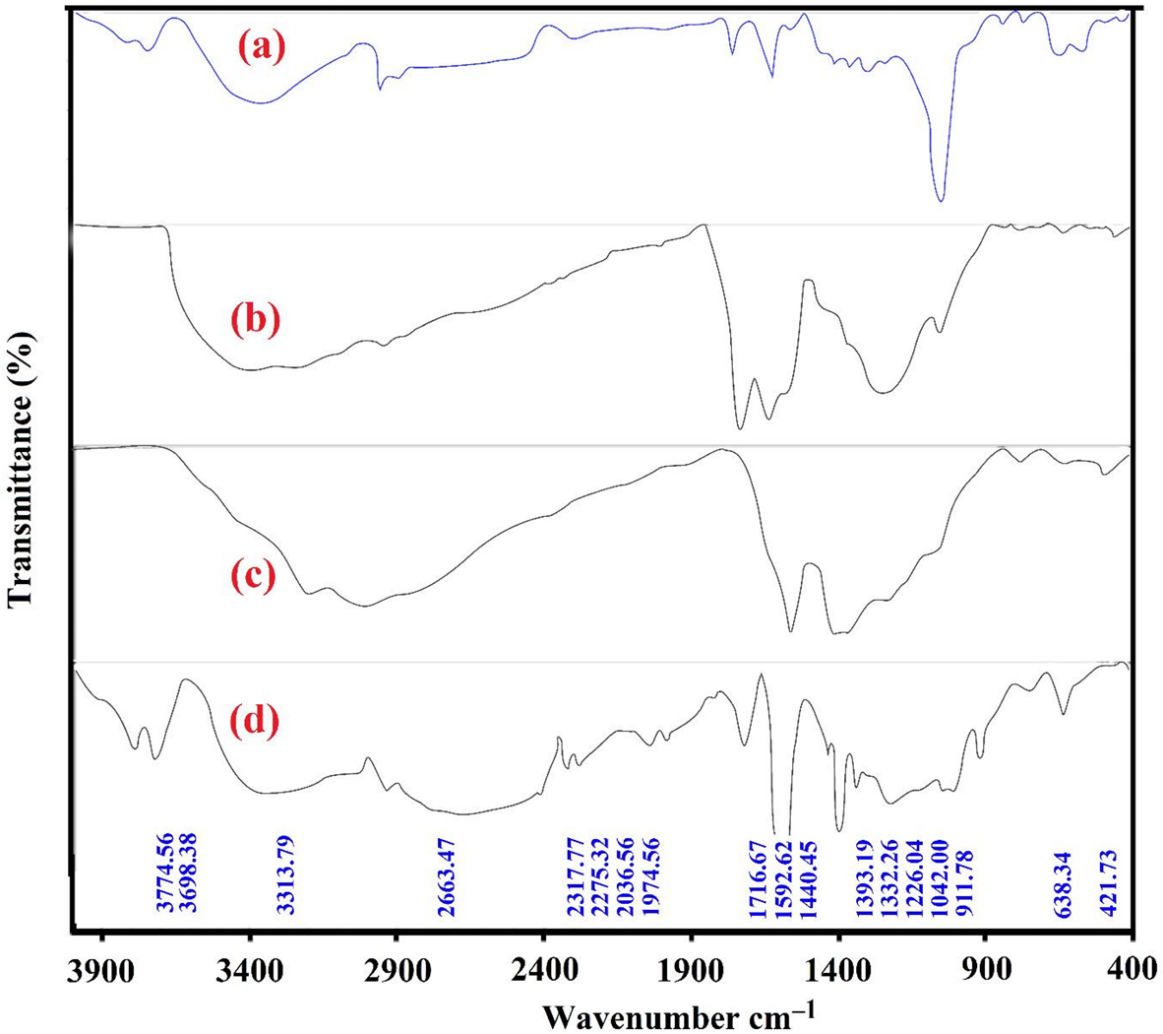
FTIR of (**a**) biochar achieved by microwave-induced H_2_SO_4_ activation; (**b**) biochar oxidized by HNO_3_ and P_2_O_5_; (**c**) amoniabiochar; (**d**) ABHG.

Fourier Transform Infrared Spectroscopy (FTIR) was employed to examine the surface chemistry of the selected samples. Figure 3 illustrates the comparison between biochar derived from orange peel exposed to microwave-induced H_2_SO_4_ activation, biochar oxidized by HNO_3_ and P_2_O_5_, and subsequently modified by ammonia (Fig. 3b–d). FTIR can be categorized into four distinct spectral areas, namely 4000–2000, 2000–1300, 1300–900, and 900–600 cm^−1^. The initial region is commonly associated with dehydration and aliphatic constituents, primarily composed of loose O–H bonds, covalently bonded OH groups, adsorbed H_2_O groups, and symmetric and asymmetric stretching in CH−, CH_2_−, or CH_3_ linkages. The second zone encompasses the primary oxygen functions, which are characterized by −NH, N–O, and C=O bonds in aldehydes, lactones, carbonyls, and carboxylic acids. The absorption in the third zone is attributed to a range of C-O single bonds, including those found in phenols, esters, hydroxyl, and ether groups. Furthermore, shoulder bands at lower wave numbers could be attributed to C–H bonds’ inherent out-of-plane bending modes, similar to those observed in benzene derivatives^63^.

The FTIR spectra of biochar derived from orange peel, which was achieved by microwave using H_2_SO_4_ activation, are illustrated in Fig. 3a. The spectra exhibit absorption peaks at 3773.52 and 3698.34 cm^−1^, indicating the existence of non-bonded hydroxyl groups and OH stretching (narrow). The observed peak at 3343.52 cm^−1^ suggests the existence of unbound or hydrogen-bonded O–H functional groups, such as those found in alcohols, carboxylic acids, and phenols, on the adsorbent’s surface^64^. The detection of faint bands at 2921.49 and 2853.17 cm^−1^ can be attributed to the stretching of aliphatic C–H bonds. The band observed at 2274.78 cm^−1^ is due to the stretching vibration of the C≡C bond. The spectral band observed around 1732.44 cm^−1^ corresponds to the stretching of C=O bonds in carboxyl, aldehyde, ketone, and lactone groups. On the other hand, the band detected at 1598.51 cm^−1^ is attributed to the vibrations of C=C bonds in aromatic rings^65^. The infrared (IR) bands at 1440.77, 1393.41, 1333.85, and 1271.28 cm^−1^ are attributed to the asymmetric stretching of the −SO_3_ groups^66^. Oxidized carbon materials exhibit an IR band around 1016.50 cm^−1^, which is attributed to the stretching of C–O or C–O–C bonds in phenols, ethers, alcohols, acids, or sulphuric acid groups (–SO_3_). According to Liang et al.^67^, the observed bands at 818.30, 735.45, and 619.49 cm^−1^ were attributed to the stretching of the S=O covalent bond. The observed band at 422.27 cm^−1^ indicates a metal oxide or an aromatic structure. Therefore, the biochar FTIR spectrum obtained by microwave-induced H_2_SO_4_ activation reveals a graphite-like structure, characterized by a high abundance of functional groups such as oxygen and sulphur.

The FTIR spectra of biochar, which were oxidized by HNO_3_ and P_2_O_5_, as depicted in Fig. 3b and subsequently modified by ammonia, as shown in Fig. 3c, exhibit distinct absorption peaks. Specifically, the bands observed at 3866.87 and 3851.51 cm^−1^ in both spectra correspond to the non-bonded hydroxyl group, represented by the OH stretch (narrow). The frequencies observed at (3382.86, 3222.73) and (3163.24, 3001.22) cm^−1^ suggest the existence of unbound or hydrogen-bonded O–H functional groups on the adsorbent’s surface. The oxidized biochar exhibits a band at 2926.57 cm^−1^, which can be attributed to the aliphatic C-H stretching. Additionally, the bands at (2112.23, 1988.04) cm^−1^ and (1709.43, 1613.64) cm^−1^ in the same chart correspond to the isothiocyanate (-NCS) stretching and C=O stretching, respectively. The spectral bands observed at around 1556.93, 1414.51, 1330.73, and (1415.07, 1362.42) cm^−1^ are associated with the nitro group (-NO_2_). A detected band at 1558.85 cm^−1^ in the ammonia-modified biochar spectrum is attributed to the asymmetric deformation of NH_3_^+^ ions. The chart showed bands at 1220.84 and 1034.80 cm^−1^, which can be attributed to C–O or C–O–C stretching. Additionally, bands at 810.80, 758.66, and 615.73 cm^−1^ were found, indicating the presence of disulfides or aromatic structures. The ammonia-modified biochar spectrum shows bands at 765.18 and 619.62 cm^−1^, which are attributed to the stretching vibration of the N–H bond. The infrared (IR) bands seen at wavenumbers (473.85, 418.54), and (499.74, 461.84, 428.92, 410.58) cm^−1^ are indicative of the existence of metal oxide or aromatic structures. According to the FTIR spectrum obtained from the oxidation of activated carbon with HNO_3_ and P_2_O_5_, the resulting chemical structure is a graphitic structure characterized by the presence of functional groups, including nitro, oxygen, and sulfur. However, the FTIR spectrum of oxidized biochar reacted with ammonia indicates a graphitic structure, characterized by distinct surface functional groups, including ammonia, nitro, and oxygen.

The FTIR spectra of ABHG obtained from aminobiochar are depicted in Fig. 3d. The spectra exhibit absorption peaks at specific resonances, namely 3774.56 and 3698.38 cm^−1^, which correspond to the non-bonded hydroxyl group and the OH stretch (narrow). The spectral profile at 3313.79 cm^−1^ suggests the existence of either unbound or hydrogen-bonded O–H functional groups on the adsorbent’s surface. The observed infrared (IR) bands at 2317.77 and 2275.32 cm^−1^ are attributed to the stretching of the isothiocyanate (C≡N) group. The infrared (IR) bands seen at 2036.56 and 1974.56 cm^−1^ are attributed to the existence of isothiocyanate (–NCS). The band at around 1716.67 cm^−1^ corresponds to the stretching of the C = O bond. The spectral bands observed at around 1592.62, 1440.45, 1393.19, and 1332.26 cm^−1^ correspond to the nitro group (–NO_2_). Bands were detected at 1226.04, 1042.00, and 911.78 cm^−1^, corresponding to stretching vibrations of C–O or C–O–C bonds. The bands observed at 638.34 and 421.73 cm^−1^ indicate either a metal oxide or an aromatic structured material.

### Adsorption of MB dye and Cr (VI) ions on ABHG

The demand for ABHG depends on its suitability for specific applications. This work focuses on the effective use of ABHG obtained from orange peels.

#### Effect of pH

**Fig. 4:**
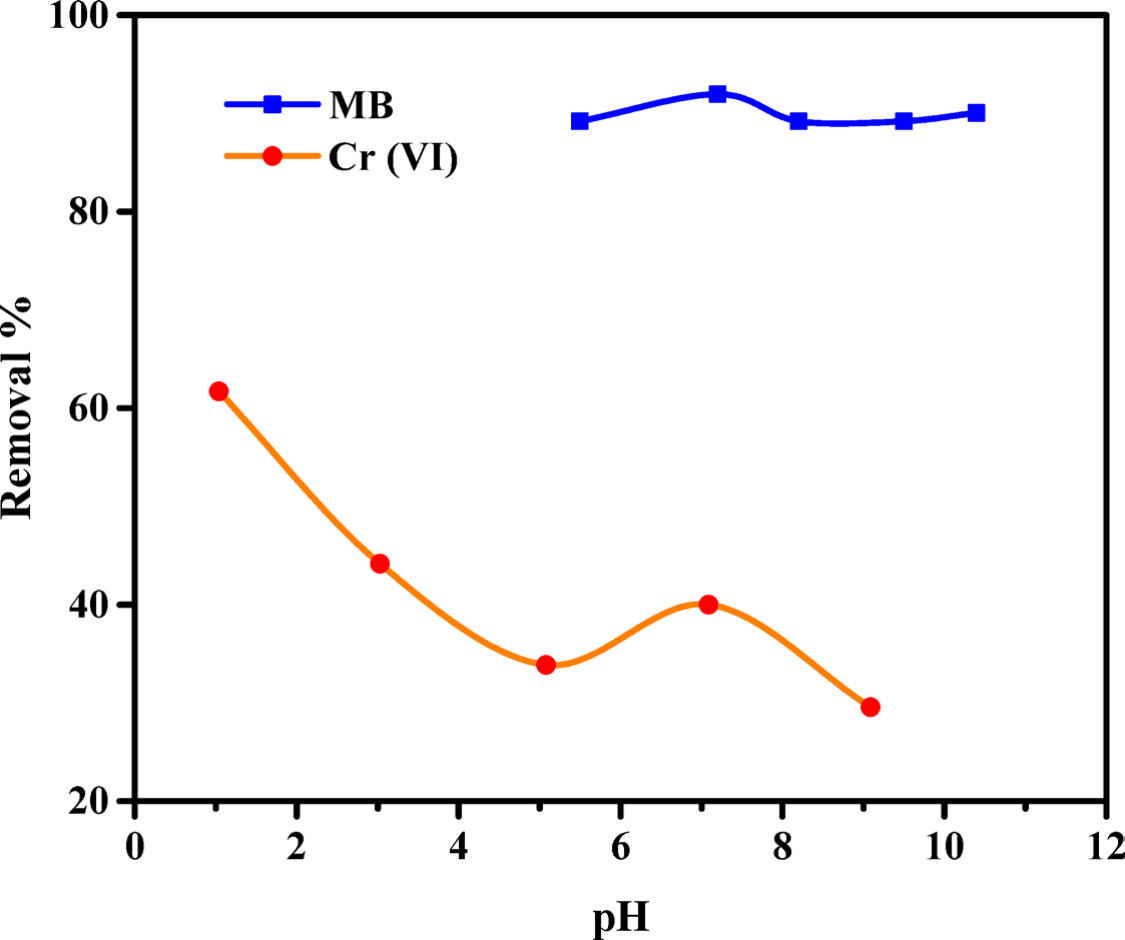
Effect of pH on the removal of MB dye and Cr (VI) ions by ABHG (*C*_0_ MB = 50 mg/L, *C*_0_ Cr (VI) ions = 75 mg/L, Adsorbent dosage for MB dye = 0.5 g/L, Adsorbent dosage for Cr (VI) ions = 0.25 g/L, Time for MB dye = 90 min, Time for Cr (VI) ions = 90 min, Temp. = 25 °C).

The pH of a pollutant solution significantly affects adsorption, as it directly influences the adsorbent’s capacity^68,69^. Experimental investigations examined the removal of MB dye or Cr (VI) ions at varying pH levels, specifically 5.5 to 10.4 for MB dye and 1.04 to 9.09 for Cr (VI) ions. For MB dye adsorption, pH values below 5.5 were not studied due to the strong protonation of amine and hydroxyl functional groups on ABHG at highly acidic conditions, which can lead to electrostatic repulsion with the cationic MB dye and thus significantly reduce adsorption efficiency. Additionally, extreme acidity can degrade the hydrogel structure, compromising its stability. The data in Fig. 4 indicate that ABHG and MB dyes exhibited higher clearance rates at acidic and basic pH conditions. The ABHG scaffold showed a peak adsorption capacity of 91.95 mg/g for MB dye at a pH of 7.2, and 185.11 mg/g for Cr (VI) ions at a pH of 1.04. According to the data presented in Fig. 4, the MB dye removal % increased from 89.17 to 91.95 as the pH was raised from 5.5 to 7.2. Subsequently, a slight drop was noted when the pH rose from 7.2 to 8.2 and 9.5. However, a little increase in the removal percentage was observed from 89.19 to 90.04 when the pH was raised from 9.5 to 10.4. This finding suggests that, under reduced pH conditions, the ABHG samples exhibited a high abundance of H^+^ ions on their surfaces. These H^+^ ions competed with the MB dye for adsorption sites on the adsorbent surface, as the MB dye inherently had cationic properties. Consequently, due to the pronounced repulsive interaction between the dyes and the adsorbent, the adsorption of dye ions on the adsorbent surfaces decreased as the pH decreased. Under elevated pH conditions, the acidic sites deprotonated, acquiring a negative surface charge. This negative charge exhibited a considerable affinity towards the cationic dye^29^.

Figure 4 shows a negative correlation between pH and the elimination % of Cr (VI) ions, with a reduction from 61.70 to 29.56%. Cr (VI) ions in aqueous solution can exist in three distinct ionic forms: HCRO_4_^−^, Cr_2_O_7_^2–^, and CrO_4_^2–^. In aqueous systems, pH largely determines the stability of these ions^36,52^. The removal efficiency of Cr (VI) ions was higher at lower pH levels, mainly due to the stronger electrostatic attraction. As the solution pH decreased, the concentration of H^+^ ions increased, neutralizing the negative charge on the adsorbent surface. Consequently, the diffusion of chromate ions to the majority of the adsorbent was enhanced^36,52^. Most Cr (VI) species exist in their monovalent forms (HCrO_4_^−^), necessitating a single exchange site and conferring a higher propensity for adsorption. The observed decline in adsorption with increasing pH can be attributed to the higher concentration of OH^−^ ions in the bulk phase, which impairs the diffusion of chromate ions. The observed decline in adsorption at elevated pH levels could be attributed to competitive interactions with chromium oxyanions.

#### Effect of adsorbent dosage

**Fig. 5:**
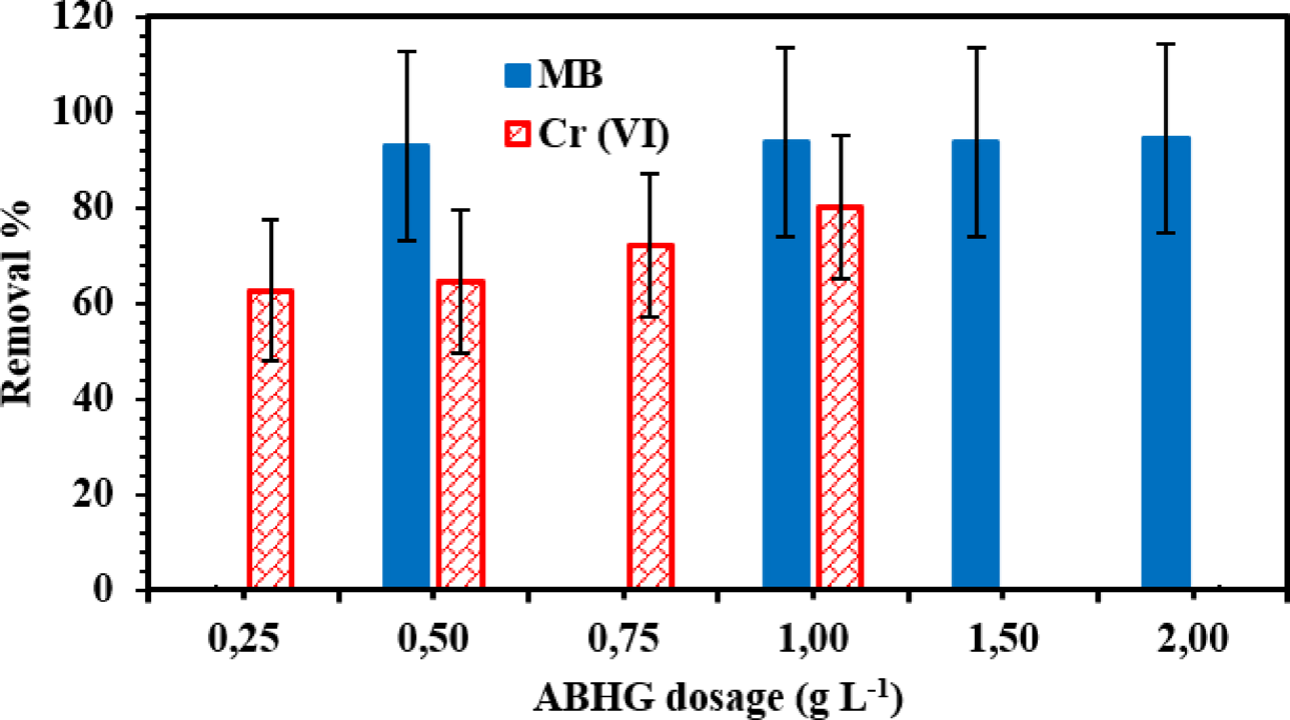
Effect of various ABHG dosages on the removal % of MB dye and Cr (VI) ions (*C*_0_ MB dye = 25 mg/L, *C*_0_ Cr (VI) ions = 25 mg/L, Time for MB dye = 90 min, pH for MB dye = 7.2, pH for Cr (VI) ions = 1.04, Time for Cr (VI) ions = 180 min, Temp. = 25 °C).

The dosage of an ABHG is a critical factor in the adsorption process as it directly influences the adsorbent’s capacity to adsorb a target substance at a specific initial concentration. The present study investigated the effect of varying adsorbent dosage on the elimination of MB dye and Cr (VI) ions while maintaining fixed values for the other parameters. Figure 5 demonstrates the effect of varying adsorbent dosages on the % adsorption of MB dye and Cr (VI) ions while employing ABHG adsorbent. The experimental results demonstrated a positive relationship between the adsorption dosage of ABHG and the elimination % across all instances. The results shown in Fig. 5 demonstrate a progressive increase in the percentage of MB dye or Cr (VI) ions removed as the adsorbent dosage increased. Although the observed rise in MB dye is modest, it is more pronounced in the presence of Cr (VI) ions. At a starting concentration of 25 mg/L, a removal efficiency of 88.31% was observed for MB dye with an adsorbent dose of 2 g/L.

In contrast, the removal efficiency for Cr(VI) ions remained at 78% while considering an adsorbent dose of 1.0 g/L. The observed higher removal percentages of MB dye or Cr (VI) ions can be attributed to the increased availability of exchangeable sites and a greater surface area at elevated adsorbent concentrations. Furthermore, increasing the adsorbent dosage while maintaining a constant dye concentration resulted in a larger accessible adsorption surface area for the dyes, thereby leading to greater dye removal^27,29^. Hence, the optimal dosages for the elimination of MB dye and Cr (VI) ions using the ABHG adsorbent were determined to be 2.0 and 1.0 g/L, respectively.

#### Effect of initial solution concentration

**Fig. 6:**
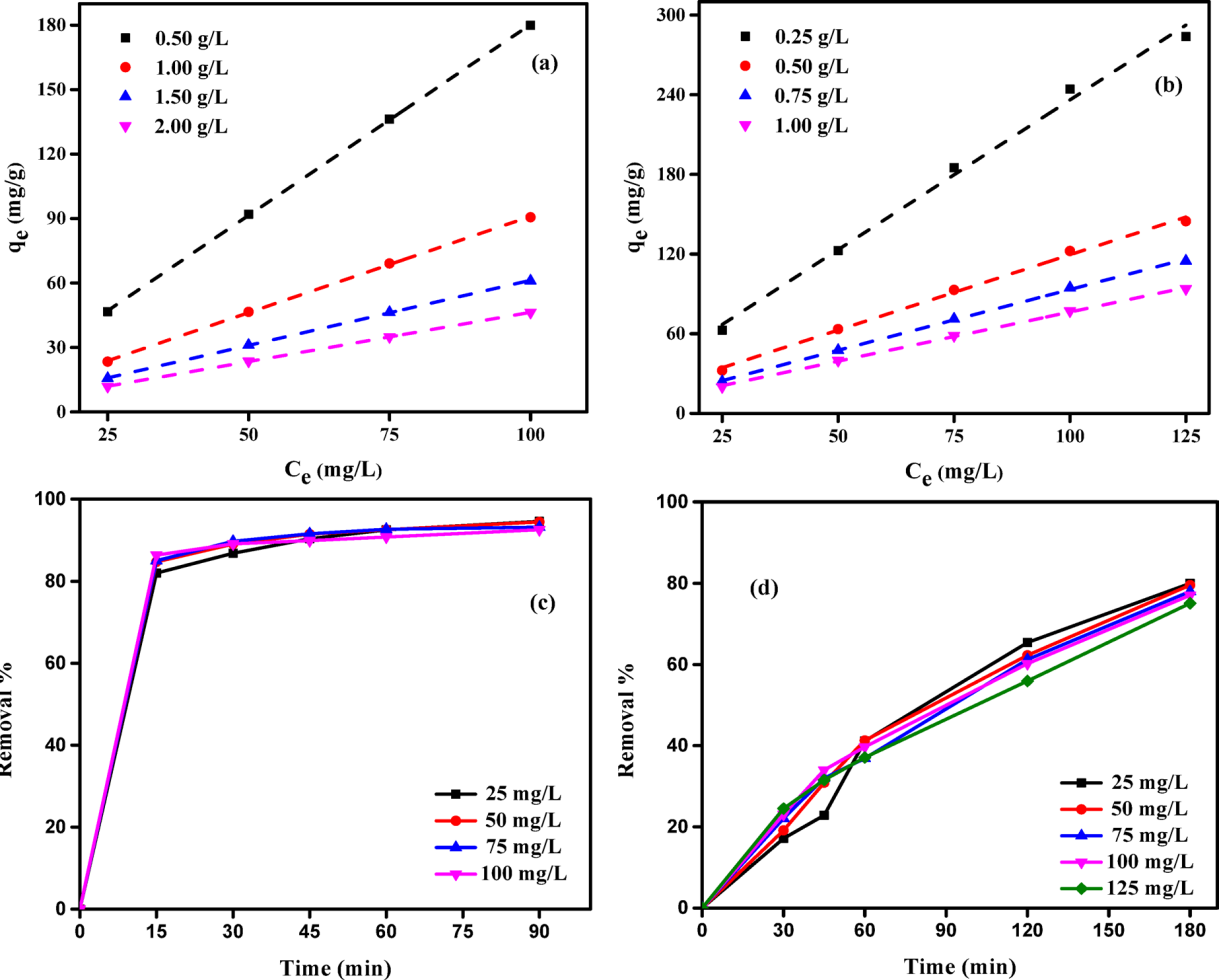
Effect of different concentrations of (**a**) MB dye and (**b**) Cr (VI) ions on adsorption capacity *q*_e_ (mg g^−1^) for each ABHG concentration and Effect of contact time on adsorption of (**c**) MB dye and (**d**) Cr (VI) ions on ABHG (Time for MB dye = 90 min, pH for MB dye = 7.2, pH for Cr (VI) ions = 1.04, Time for Cr (VI) ions = 180 min, Temp. = 25 °C).

An investigation was conducted to examine the impact of varying initial concentrations of MB dye or Cr (VI) ions on adsorption capacity. To investigate the impact of various initial concentrations (25, 50, 75, 100, and 125 mg/L) and ABHG dosages (0.50–2.00 g/L for MB dye and 0.25–1.00 g/L for Cr (VI) ions), the experiments were achieved with a contact period (90 min for MB dye and 180 min for Cr (VI) ions), and a pH (7.2 for MB dye and 1.04 for Cr (VI) ions). The results of this investigation are illustrated in Fig. 6a and b. The adsorption capacity of ABHG showed a positive correlation with increasing initial solution concentration. The equilibrium adsorption capacity (*Q*_e_, mg/g) value rose from 46.66 to 179.93 mg/g when the ABHG dosage was 0.50 g/L, and the starting dye concentration varied between 25 and 100 mg/L. The equilibrium adsorption capacity, *Q*_e_ (mg/g), rose from 23.39 to 90.59, 15.63 to 61.04, and 11.83 to 46.30 mg/g, respectively, when the ABHG doses were 1.00, 1.50, and 2.00 g/L and the initial MB dye concentration varied between 25 and 100 mg/L. Increasing the dosage of ABHG from 0.5 to 2.0 g/L, while maintaining a starting MB dye concentration of 100 mg/L, resulted in a reduction of the maximum adsorption capacity of MB dye from 179.93 to 46.30 mg/g (Fig. 6a).

In the case of Cr (VI) ions, the value of *Q*_e_ (mg/g) rose from 62.67 to 283.78 mg/g when the ABHG dose was 0.25 g/L and the starting concentration of Cr (VI) ions ranged from 25 to 125 mg/L (Fig. 6b). The adsorption capacity at equilibrium, *Q*_e_ (mg/g), rose from 32.29 to 144.71, 24.00 to 114.86, and 20.00 to 93.86 mg/g, respectively, when the ABHG doses were 0.50, 0.75, and 1.00 g/L, and the starting Cr (VI) ions concentration ranged from 25 to 125 mg/L. When the dosage of the ABHG adsorbent was raised from 0.25 to 1.0 g/L, while the initial Cr (VI) ions concentration was kept at 125 mg/L, it was observed that the maximum adsorption capacity of the Cr (VI) ions diminished from 283.78 to 93.86 mg/g. It was determined that the ABHG adsorbent had a higher Cr (VI) ion removal capacity than the MB dye at all initial concentrations studied. This indicates that the adsorption process strongly correlated with the solution’s initial concentration. The observed outcomes can be ascribed to insufficient active sites to accommodate the elevated starting concentrations of MB dye or Cr (VI) ions. Adsorption sites exhibit a higher adsorption rate for accessible solutes at lower concentrations.

#### Effect of contact time using ABHG

**Fig. 7:**
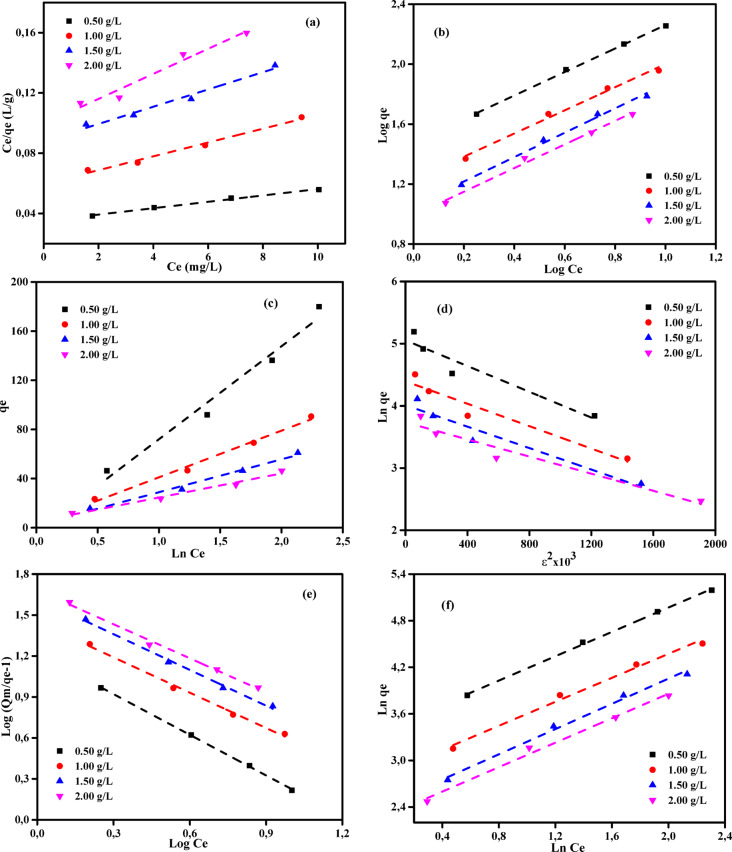
(**a**) LIM, (**b**) FIM, (**c**) TIM, (**d**) DRIM, (**e**) GIM, (**f**) HIM profiles for MB dye of initial concentration (25–100 mg/L) on ABHG doses (0.50–2.00 g/L) at 25 °C, contact time: 90 min.

The measure of contact time is an intrinsic characteristic of all transfer phenomena, including adsorption. Figures 6c and d illustrate the impact of the contact time of the MB dye and Cr (VI) ions on the removal rates of ABHG over 90 min for the MB dye and 180 min for Cr (VI) ions. Figures 6c and d demonstrate that the equilibrium time is influenced by adsorbate concentration, and this relationship varies with the initial concentration and type of adsorbate. Figure 6c shows that a significant proportion of the MB dye is removed efficiently within 15 min. The removal efficiency of MB dye was 82.01% at an initial concentration of 25 mg/L and 86.40% at an initial concentration of 100 mg/L. Following this, there was a slow, marginal increase in the elimination rate until it reached equilibrium after 90 min, at which point it stabilized at 94.63% and 92.60%, respectively. If a similar comparison is conducted for the Cr (VI) ions, Fig. 7d illustrates a steady increase in Cr (VI) ion removal over 180 min. At a starting concentration of 25 mg/L of Cr (VI) ions, the removal efficiency was 17.14%. Similarly, at a beginning concentration of 125 mg/L, the removal efficiency achieved was 24.51% within the initial 30-min period. Subsequently, the removal rate gradually increased until it reached equilibrium after 180 min, at which point it stabilized at 80% and 75.09%, respectively. At lower concentrations, an increased ratio of accessible surface area to the initial concentration of Cr (VI) ions resulted in greater independence of the elimination process from the initial concentration. However, at elevated concentrations, this ratio decreased. The elimination rate was found to depend on the starting concentration^35,69^.

### Adsorption isotherm studies

**Fig. 8:**
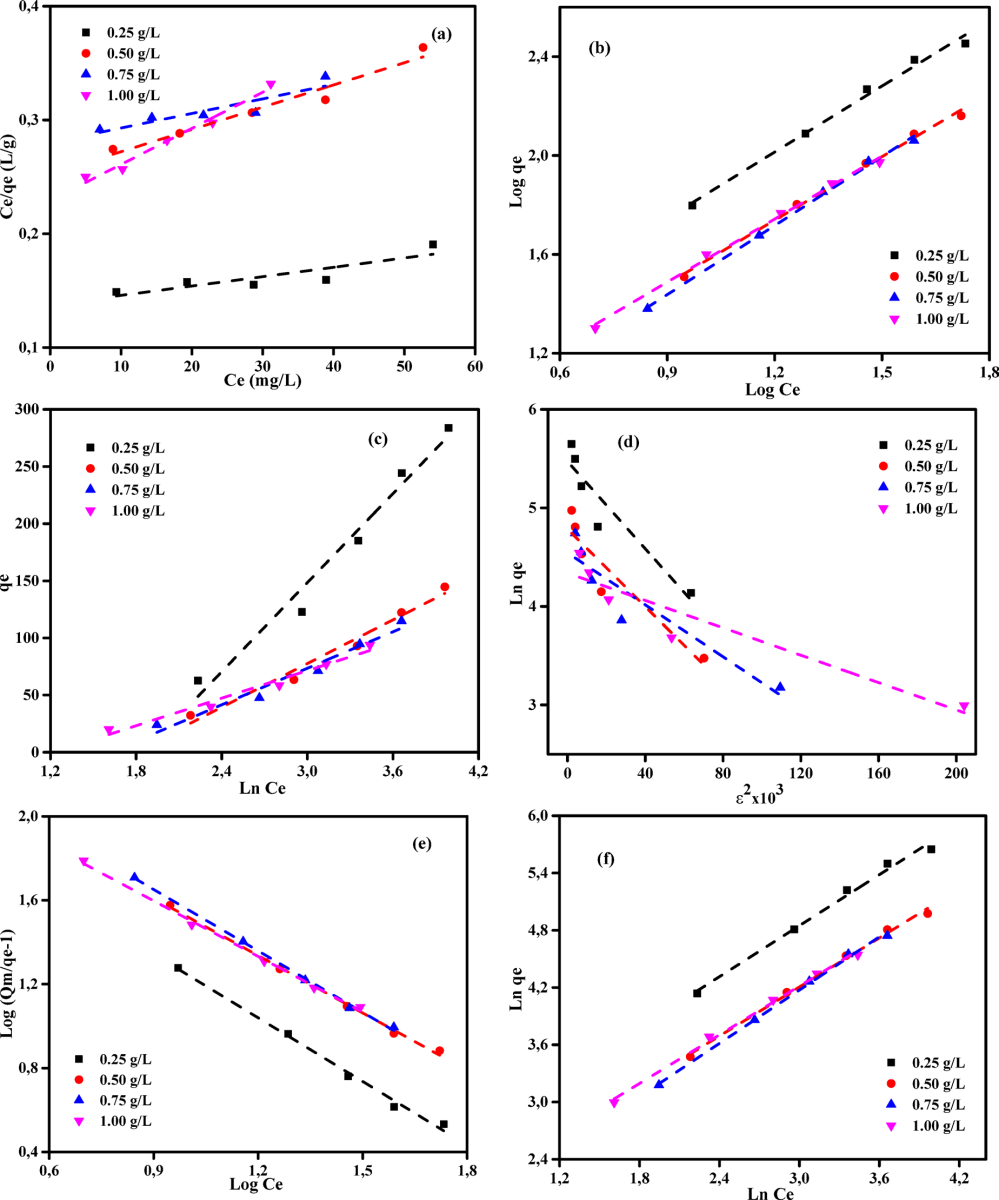
(**a**) LIM, (**b**) FIM, (**c**) TIM, (**d**) DRIM, (**e**) GIM, (**f**) HIM profiles for Cr (VI) ions of initial concentration (25–125 mg/L) on ABHG doses (0.25–1.00 g/L) at 25 °C, contact time: 180 min.

The adsorption isotherm is a significant analytical tool used to elucidate the mechanisms that control the retention or migration of substances from aqueous environments or porous materials to a solid phase under constant conditions^70,71^. The state of equilibrium in adsorption is achieved when the quantity of pollutant adsorbed is equivalent to that of a pollutant that remains in the solution. The equilibrium is achieved when the phase containing the adsorbate interacts with the adsorbent for a sufficient period, resulting in a reduction of the adsorbate concentration in the bulk solution to a stable state corresponding to the concentration at the interface^72,73^. The utilization of the mathematical relationship between the solid phase and the concentration of the leftover liquid, as illustrated and empirically demonstrated by Ncibi^74^, is a widely employed approach. The link above is significant for the conceptualization, development, and practical use of removal methodologies. According to El Nemr et al.^56^, a comprehensive understanding of the adsorption mechanism, surface characteristics, and adsorbent affinities can be achieved by examining thermodynamic theories and physicochemical factors associated with specific substances. The assessment of experimental data has been facilitated by the development of several adsorption isotherm models, including the Langmuir (LIM), the Freundlich (FIM), Temkin (TIM), Dubinin and Radushkevich (DRIM), Generalized Isotherm (GIM), and Halsey isotherm models (HIM)^56,75,76^. Tables S1-S2 and Figs. 7–8 present the results of an investigation into six adsorption isotherms (LIM, FIM, TIM, DRIM, GIM, and HIM) for the adsorption of MB dye and Cr (VI) ions on ABHG media.

The Langmuir monolayer adsorption process, as described by Langmuir in 1916^75^, entails the homogeneous partitioning of a solute from a liquid solution onto a surface. The present procedure does not involve the migration of the adsorbate onto the respective surface plane. The surface exhibits a predetermined quantity of identical locations where adsorption occurs^77^. The LIM methodology was chosen to determine the maximum adsorption capacity (*Q*_m_, mg/g) required to achieve full monolayer coverage on the sorbent’s surface. The equation representing LIM is denoted as Eq. 4.

4 $$\frac{{C}_{e}}{{q}_{e}}=\frac{1}{{K}_{L}}{Q}_{m}+\frac{1}{{Q}_{m}}\times {C}_{e}$$

where *C*_e_ (mg/L) represents the concentration of adsorbate in solution at equilibrium, *q*_e_ (mg/g) represents the adsorption capacity at equilibrium, *K*_L_ (L/mg) is the LIM adsorption constant, and *Q*_m_ (mg/g) is the maximum adsorption capacity at monolayer coverage.

The LIM data are presented in Tables S1-S2, while the LIM plots of the MB dye and Cr (VI) ions are depicted in Figs. 7a and 8a, correspondingly. In this study, the ABHG adsorbent demonstrated robust linear correlations (*R*^2^ = 0.965–0.994) and (*R*^2^ = 0.771–0.981) with the LIM, suggesting its efficacy in the elimination of MB dye and Cr (VI) ions, respectively. Regarding monolayer capacities (*Q*_m_), the MB dye and Cr (VI) ions exhibit capacities of 476.19 and 1250 mg/g, respectively. The saturation capacity was not reached under the tested conditions. The intersection point of the *C*_e_/*q*_e_ vs. *C*_e_ plot and the slope of the plot, as depicted in Figs. 7a and 8a, were employed to determine the values of 1/*Q*_m_*K*_L_ and 1/*Q*_m_ for the LIM component, respectively. The *K*_L_ values of 0.06 to 0.084 L/mg for MB dye and 0.005 to 0.014 L/mg for Cr (VI) ions provide compelling evidence for the adsorption of adsorbates on ABHG. These findings suggest that the LIM is relevant to MB dye in ABHG, but its applicability to Cr(VI) ion ABHG is limited. A thorough analysis of the ABHG adsorbent showed that the absorption of both MB dye and Cr (VI) ions occurred just inside a singular layer on its surface.

#### Freundlich isotherm

The initial equation introduced for the investigation of the adsorption process is the Freundlich-Isotherm equation (FIM)^78^. Given its exponential nature, the FIM formulation posits that the adsorbate’s concentration on the adsorbent’s surface increases in direct proportion to the adsorbate’s concentration. The mathematical expression was represented as seen in Eq. (5).

5 $$Ln {q}_{e }=\mathrm{ln}{K}_{F}+\frac{1}{n}\mathrm{ln}{C}_{e}$$

where *n* and* K*_F_ (mg^1–1/n^ g^−1^ L^1/n^) denote the Freundlich constants, representing the intensity and capacity of adsorption, respectively.

Data on the FIM parameters are presented in Tables S1 and S2. Additionally, Figs. 7b and 8b show the FIM plots for the MB dye and Cr (VI) ions, respectively. The intersection point of the ln (*q*_e_) vs. ln (*C*_e_) plot, as depicted in Figs. 7b and 8b, and the slope of the plot, respectively, yield the logarithmic values for *K*_F_ and 1/*n* of the FIM. The binding energy, denoted as *K*_F_ (L/g), is a fundamental parameter in the FIM equation that quantifies the extent to which MB dye or Cr (VI) ions are eliminated from the ABHG at a given equilibrium concentration. According to Fan et al.^79^, when 1/*n* is less than 1, the adsorbent has a higher binding affinity for the adsorbate. Hence, in cases where the ratio 1/n is below 1, eliminating both MB dye and Cr (VI) ions using the ABHG adsorbent can be classified as a physical phenomenon. The 1/*n* values in Tables S1 and S2 are below 1, suggesting that the ABHG adsorbent effectively adsorbs both MB dye and Cr (VI) ions. The linear relationship between log(*q*_e_) and log(*C*_e_) accurately determines the values of the FIM correlation coefficients (Figs. 7b and 8b). The ABHG adsorbent exhibited greater Freundlich correlation values (*R*^2^ = 0.991–0.999) and (*R*^2^ = 0.991–0.948) compared to the Langmuir correlation coefficient for the MB dye and Cr (VI) ions, respectively.

#### Temkin isotherm

The Temkin isotherm model (TIM)^80^ proposes a theory of indirect interactions between adsorbate and adsorbent. The Temkin isotherm theory posits that interactions between the adsorbent and adsorbate result in a linear decrease in the heat of adsorption per molecule in the layer as coverage increases. The adsorption process is characterized by a consistent distribution of binding energies, reaching a maximum binding energy. The Temkin isotherm model can be explained using Eq. (6), which can be simplified to the linear relation form in the following Eq. (7)^56^:

6 $${Q}_{e}=\frac{RT}{{B}_{T}}\mathrm{ln}({A}_{T}{C}_{e})$$

7 $${Q}_{e}=\frac{RT}{{B}_{T}}\mathrm{ln}\left({A}_{T}\right)+\frac{RT}{{B}_{T}}\mathrm{ln}({C}_{e})$$

where *A*_T_ (L/mg) is the Temkin isotherm constant, *B*_T_ (J g mol^−1^ mg^−1^) represents the adsorption energy (heat of adsorption) variation factor, *R* (8.314 J mol^−1^ K^−1^) represents the universal gas constant, and *T* (K) represents the absolute temperature.

The TIM parameters (AT and BT) can be determined from the linear correlation between *q*_e_ and ln*C*_e_, as depicted in Fig. 7c and 8c. The graph exhibits a slope of AT (g/L), while the point of intersection corresponds to BT. Tables S1 and S2 summarize the TIM constants. The obtained correlation coefficients (*R*^2^ = 0.983–0.991) and (*R*^2^ = 0.964–0.975) for the analysis of the impact of temperature change on the elimination of MB dye and Cr (VI) ions using the ABHG adsorbent, respectively, were found to be significantly high. These results show that the model employed in the study is appropriate for the investigation. The heat of adsorption (BT), the energy released during the interaction between the adsorbate and the adsorbent, plays a crucial role in the adsorption of MB dye and Cr (VI) ions by the ABHG adsorbent. Upon analysis of the acquired data, it was observed that the absorption temperatures were significantly low, indicating that the adsorption process was predominantly physisorption.

#### The Dubinin–Radushkevich (D-R) isotherm

The Dubinin-Radushkevich isotherm model (DRIM) quantifies the apparent free energy of porosity and the adsorption properties^81–83^. The DRIM does not rely on the assumptions of surface homogeneity or a constant sorption potential. It is frequently expressed in Eq. (8), and its linear representation is shown in Eq. (9).

8 $${q}_{e}={Q}_{m}\mathrm{exp}(-K{\varepsilon }^{2})$$

9 $$ln{q}_{e}=ln{Q}_{m}-{K\varepsilon }^{2}$$

where *Q*_*m*_ (mg/g) represents the theoretical saturation capacity, *K* (mol^2^ (kJ^2^)^−1^) denotes a constant related to the adsorption energy, and ε represents the Polanyi potential, which can be determined using Eq. (10)^56^:

10 $${\varepsilon}=\mathrm{RT}\times \mathrm{ln}\left(1+\frac{1}{{\mathrm{C}}_{e}}\right)$$

The slope of the linear relationship between *q*_e_ and *ε*^2^ provides the value of *K* (mol^2^ (kJ^2^)^−1^)), whereas the intercept represents the adsorption capacity, *Q*_m_ (mg/g). The calculation of the mean free energy of adsorption (E) involved determining the change in free energy upon transferring one mole of ion from the bulk solution to the sorbent surface. This calculation used the K value in the following Eq. (11)^56^.

11 $$E=1/\sqrt{(2K)}$$

The Dubinin-Radushkevich isotherm model (DRIM) is used to analyze the equilibrium data and determine whether the elimination of MB dye and Cr(VI) ions from the ABHG adsorbent is dominated by chemical or physical processes. Within this theoretical framework, the Polanyi potential theory posits that the removal process persists until the pores reach their maximum capacity. Tables S1 and S2 present the correlation coefficients and DRIM constants derived from the adsorption of MB dye or Cr (VI) ions onto the ABHG surface at various doses. The apparent energy (*E*) magnitude estimates the specific adsorption type. The determination of adsorption type can be achieved by analyzing the evident energy (*E*) values, which can be categorized as follows: *E* < 8 kJ/mol indicates “physical adsorption”, *E* < 16 kJ/mol signifies “ion exchange”, and *E* > 16 kJ/mol indicates “chemical adsorption”^84,85^. Upon examination of the estimated binding energy (*E*) values presented in Tables S1 and S2, it becomes evident that all dosages of ABHG adsorbent exceed 16 kJ/mol for the removal of MB dye. Conversely, these values are below 8 kJ/mol for the removal of Cr (VI) ions. Consequently, it can be inferred that the adsorption of MB dye on ABHG adsorbent is primarily attributed to chemical adsorption for MB dye removal, while the removal of Cr (VI) ions is attributed to physical adsorption. The achieved correlation coefficient (*R*^2^) values for eliminating MB dye and Cr (VI) ions using the ABHG adsorbent at various dosages in the DRIM range are 0.913–0.942 and 0.881–0.888, respectively. These values are more consistent with the experimental data for most adsorbent dosages (Figs. 7d and 8d; Tables S1 and S2). Furthermore, the ABHG adsorbent exhibits a maximum monolayer capacity of *Q*_m_ of 476.19 mg/g for MB dye and 1250.00 mg/g for Cr (VI) ions. These findings suggest that the DRIM may have limited applicability compared to the LIM and FIM models^56^.

#### Generalized isotherm equation

Generalized isotherm equation given in the linear form by Eq. (12)^56^:

12 $$log\left[\frac{{Q}_{\mathrm{m}}}{{q}_{\mathrm{e}}}-1\right]=log{K}_{\mathrm{G}}-{N}_{\mathrm{b}}log{C}_{\mathrm{e}}$$

where *N*_b_ is the cooperative binding constant, *K*_G_ (mg/L) is the saturation constant, and *Q*_m_ (mg/g) represents the maximum adsorption capacity of the adsorbent (as determined from LIM). *C*_e_ (mg/L) and *q*_e_ (mg/g) denote the equilibrium concentrations of MB dye or Cr (VI) ions in the liquid and solid phases, respectively. A graph displayed in Figs. 7e and 8e shows the relationship between log [(*Q*_*m*_*/q*_e_)–1] and log *C*_e_. The intercept corresponds to log *K*_G_, while the slope corresponds to *N*_b_ constants. The parameters associated with each isotherm were determined by linear regression, and the correlation coefficient (*R*^2^) was computed. Tables S1 and S2 compile the acquired parameters and their corresponding *R*^2^ values..

#### The Halsey isotherm model

The Halsey isotherm model (HIM) can be characterized by utilizing heteroporous materials and multilayer adsorption techniques^86,87^. The HIM is denoted by Eq. (13):

13 $$ln{q}_{e}=\frac{1}{n}lnk+\frac{1}{n}ln{C}_{e}$$

The values of *n* and *k* can be ascertained by analyzing the data derived from the linear function plot of *ln q*_e_ vs *ln C*_e_, as depicted in Figs. 7f and 8f. The numbers above correspond to the adsorption constants of HIM systems. Based on the satisfactory fit of the model to the equilibrium data, it can be concluded that the adsorbent exhibits non-homogeneity. Based on the analysis of Figs. 7f and 8f, it can be observed that the MB dye and Cr (VI) ions, when adjusted for the correction factor, exhibit a higher degree of suitability in fitting the data obtained from LIM, TIM, and DRIM across all adsorbent dosages. The statistical analysis conducted using the HIM revealed a significant correlation (*R*^2^ = 0.991–0.999) and (*R*^2^ = 0.991–0.998) between MB dye and Cr (VI) ions (Tables S1 and S2). The adsorption of many layers within the pores yielded significant *R*^2^ values when the adsorption outcomes were analyzed using the FIM and HIM.

### Adsorption kinetic studies

**Fig. 9:**
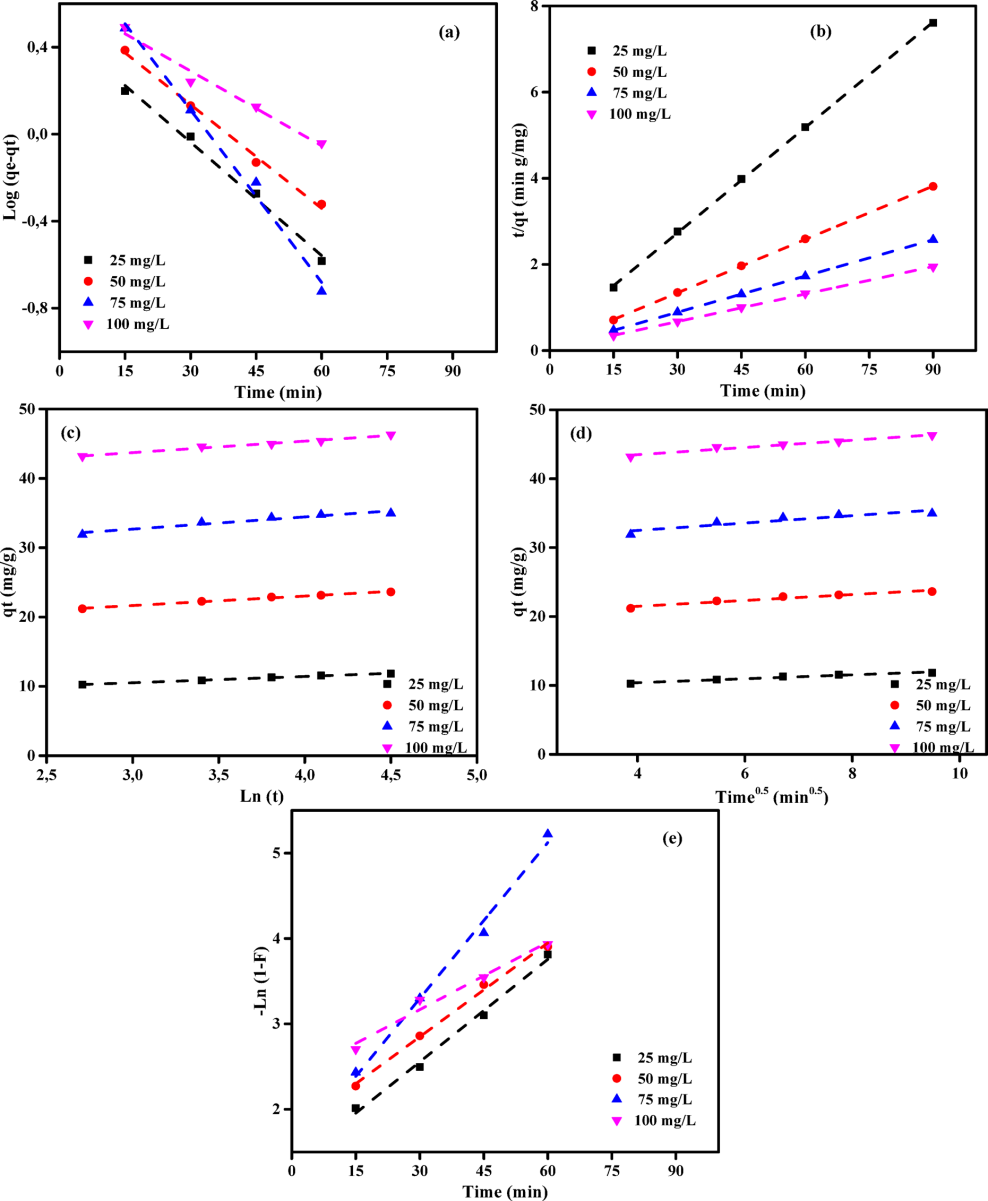
The plot of (**a**) PFOM, (**b**) PSOM, (**c**) EM, (**d**) IPDM, and (**e**) FDM of adsorption of MB dye by ABHG adsorbent (*C*_0,_ dye = (25–100 mg/L), C_0_, ABHG = (2.0 g/L), Temp. = 25 °C).

**Fig. 10:**
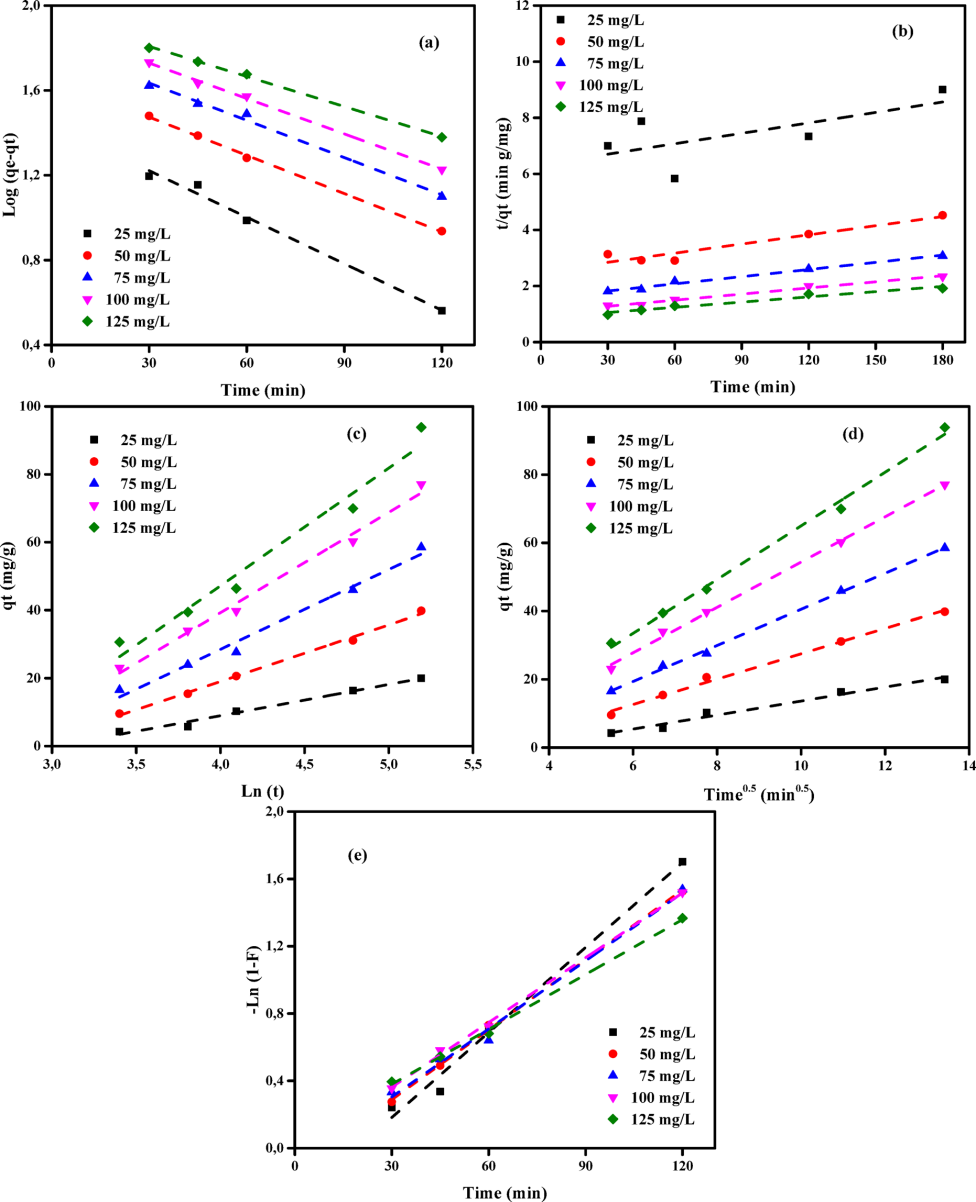
The plot of (**a**) PFOM, (**b**) PSOM, (**c**) EM, (**d**) IPDM, and (**e**) FDM of adsorption of Cr (VI) ions by ABHG adsorbent (*C*_0,_ Cr (VI) = (25–125 mg/L), *C*_0_, ABHG = (1.0 g/L), Temp. = 25 °C).

A comprehensive examination of many models, encompassing diffusion control, mass transfer, and chemical reaction, was undertaken to examine the kinetics of adsorption. This study aimed to examine the kinetics of adsorption of MB dye and Cr (VI) ions onto ABHG. The objective was to ascertain the most favourable operational parameters for a large-scale batch manufacturing procedure. A comprehensive understanding of the kinetic parameters is crucial for predicting adsorption rates and providing robust evidence for advancing and simulating adsorption processes. Hence, the kinetic models of pseudo-first-order (PFOM)^88^, pseudo-second-order (PSOM)^89^, Elovich (EM)^90,91^, intraparticle diffusion (IDM), and film diffusion (FDM)^92,93^ were utilized in the examination of the elimination of MB dye or Cr (VI) ions onto ABHG. The degree of agreement between the theoretical values predicted by the models and the empirical data was assessed using the *R*^2^ (correlation coefficient), as depicted in Figs. 9 and 10 and Tables S3-S6.

#### Pseudo-first-order model (PFOM)

The Lagergren PFOM^94^ theoretical framework was employed to estimate the adsorption rate constant, an initial model that characterizes the adsorption rate by accounting for adsorption capacity. Equation (14) denotes the PFOM kinetic model.

14 $$\mathrm{log}\left({q}_{\mathrm{e}}-{q}_{\mathrm{t}}\right)=\mathrm{log}\left({q}_{\mathrm{e}}\right)-\frac{{k}_{1}}{2.303}t$$

The variables *q*_t_ and *q*_e_ (mg/g) denote the volumetric quantities of ions adsorbed at time *t* and equilibrium, respectively. The variable *k*_*1*_ (min^−1^) represents the rate constant associated with the adsorption process of PFOM. The values of *k*_1_ and *q*_e_ were found through the computation of the slope and intercept components of the logarithmic plots of (*q*_e_ ≦ *q*_t_) against *t*, as depicted in Figs. 9a and 10a. The kinetic models in Tables S3 and S5 exhibit correlation coefficients (*R*^2^) ranging from 0 to 1. The adequacy of the model is directly correlated with the correlation coefficient (*R*^2^) value approaching unity. The observed *R*^2^ values, all exceeding 0.900, indicate a robust relationship between the estimated and experimental *q*_e_ values. The data analysis presented in Tables S3 and S5 indicates that the PFOM equation is a suitable model for describing the adsorption of Cr (VI) ions onto ABHG. The findings illustrated in Tables S3 and S5 indicate that there is no consistent trend of either an increase or decrease in *R*^2^ values as the concentration of ABHG increases within the range of 0.5 to 2.0 g/L for MB dye and from 0.25 to 1.0 g/L for Cr (VI) ions.

#### Pseudo-second-order model (PSOM)

This study used the PSOM method to evaluate the effectiveness of the ABHG adsorbent in removing MB dye and Cr (VI) ions. The PSOM hypothesis posits that the rate of solute adsorption is directly proportional to the number of accessible adsorption sites on the adsorbent. Furthermore, the amount of solute on the adsorbent surface has a significant effect on the reaction rate. The relationship between the *q*_e_*-q*_t_ and the number of active sites on the adsorbent has been experimentally demonstrated^95,96^. The PSOM can be mathematically expressed using Eq. (15):

15 $$\left(\frac{t}{{q}_{t}}\right)=\frac{1}{{k}_{2}{q}_{e}^{2}}+\frac{1}{{q}_{e}}(t)$$

where *k*_2_ (g mg^−1^ min^−1^) represents the equilibrium rate constant for PSOM adsorption^97^. Assuming the PSOM is suitable, the graph of *t/q*_t_ vs *t* demonstrates a linear correlation. The values of the variables *k*_2_ and *q*_e_ can be determined from the slope and intercept of the line, respectively. The correlations above are depicted in Figs. 9b and 10b. The PSOM curve of the ABHG for the elimination of the MB dye and Cr (VI) ions is demonstrated in Figs. 9b and 10b, respectively. Tables S3 and S5 present the PSOM constant (*k*_2_) values, the theoretical and experimentally derived *q*_e_ values, and the accompanying *R*^2^ metrics. Based on an analysis of Tables S3 and S5, the PSOM shows *R*^2^ values that closely approximate 1. The experimental *q*_e_ values show a high degree of agreement with the calculated *q*_e_ values across all investigated initial MB concentrations. The PSOM is the optimal kinetic model for characterizing the behavior of MB dye. However, the absence of comparable findings with Cr (VI) ions may be attributed to the relatively low *R*^2^ values observed at specific doses.

#### Elovich kinetic model (EM)

The investigation of MB dye and Cr (VI) ion removal by the ABHG adsorbent was conducted using the Elovich Model (EM). The EM is a valuable tool for predicting the energy needed for the activation or deactivation of a system, as well as the motion of mass and particles on the physical surface. The approach above has been effectively employed in wastewater treatment despite its initial development for gaseous systems. Based on the theoretical frameworks proposed by Cheung et al.^98^ and Dotto and Pinto^99^, it can be observed that an increase in the quantity of solute deposited results in a significant drop in the rate of solute adsorption, with an exponential decline. EM is employed to examine the presence of diverse active sites on the adsorbent material. Equation (16) characterizes the representation of EM:

16 $${q}_{\mathrm{t}}=\frac{1}{\beta }\mathrm{ln}\left(\alpha \beta \right)+\frac{1}{\beta }\mathrm{ln}(t)$$

where *α* (mg g^−1^ min^−1^) and *β* (g mg^−1^) denote the initial sorption rate constant and the surface coverage, as well as the activation energy for chemisorption, respectively. Determining the values of these constants can be achieved by computing the slope and intercept of the plot of *q*_t_ versus *ln t*, employing the model equation as depicted in Figs. 9c and 10c. The Elovich Model (EM) constants were established by computing the intercept and slope of the Figs. 9c and 10c, respectively. The calculated results are displayed in Tables S4 and S6. The *R*^2^ correlation coefficients for the adsorption of MB dye and Cr (VI) ions onto ABHG range from 0.921 to 0.997 and 0.956 to 0.999, respectively. These values suggest that the model applies to the experimental results for the adsorption of MB and Cr (VI) ions onto ABHG. Upon analyzing the *R*^2^ values, it is evident that the *R*^2^ values of EM show a slight increase compared to PFOM, while maintaining lower values than PSOM, as indicated in Tables S3-S6. The results in Tables S4 and S6 demonstrate that chemical adsorption significantly controls the adsorption rates of MB dye and Cr (VI) ions on the ABHG adsorbent.

#### Intra-particle diffusion model (IPDM)

The adsorption technique employed for removing pollutants using a solid phase material can be deduced to comprise four sequential stages: (1) The transfer of pollutants from a solution to the uppermost layer of a solid phase occurs via bulk diffusion. (2) Pollutant traverses the boundary layer using film diffusion, originating from the surface of the solid phase. (3) The transfer of pollutants occurs through either intraparticle or pore diffusion, including the interaction between the surface of the solid phase and the inner pores of its particles. (4) The pollutant undergoes adsorption at an active site located on the surface of the solid phase by a chemical process, including chelation, ion exchange, or complexation. In general, the mechanisms by which mass is transferred between the liquid phase and the control pollutant adsorption in the IPDM have been investigated by Kargi and Cikla^100^. Isothermal plug-flow diffusion (IPFD) can be used as the rate-controlling mechanism in an experimental configuration employing a batch approach and vigorous agitation^101^. The examination of the IPDM also encompassed the utilization of Eq. (17) as proposed by Annadurai et al.^102^.

17 $${q}_{t}={K}_{diff}{t}^{0.5}+C$$

where *K*_*diff*_ represents the rate constant of IPDM (mg g^−1^ min^1/2^) (Figs. 9d and 10d). The concept put forward by Weber and Morris^103^ posits that the adsorption process is governed by the intraparticle diffusion step, as indicated by the intersection of the lines representing *q*_t_ and the square root of time (*t*) in Figs. 9d and 10d at the vector origin. However, when the lines do not intersect the origin, it is widely accepted that the elimination process is mostly governed by the Film diffusion model (FDM), especially when the *C* value is high. The study investigated the efficacy of removing MB dye or Cr (VI) ions from ABHG samples using different adsorbent dosages and varying initial concentrations of MB dye or Cr (VI) ions. The images presented in Figs. 9d and 10d illustrate the Webber-Morris^104^ adsorption lines for MB dye and Cr (VI) ions, respectively. The *K*_dif_ and *C* values presented in Tables S4 and S6 were obtained by analyzing the slope and intercept of the plot of *q*_t_ versus *t*^0.5^. The linear trends depicted in Figs. 9d and 10d, which illustrate the various concentrations of the adsorbent, show no intersection at the origin for both MB dye and Cr (VI) ions. This can be attributed to the observed high *C* intersection. This assertion can be substantiated by the observation that FDM significantly influences the rate of absorption of both MB dye and Cr (VI) ions into the ABHG adsorbent. As depicted in Figs. 9e and 10e observed that the absorption rate progressively increases over time. The intra-particle diffusion rate constant, denoted as *K*_dif_, exhibited a range of 0.29 to 3.02 mg g^−1^ min^−1/2^ for the loading of MB onto ABHG. In the case of Cr (VI) deposited onto ABHG, the intra-particle diffusion rate constant, denoted as *K*_dif_, exhibited a range of 2.05 to 21.80 mg g^−1^ min^−1/2^ for Cr (VI) ions. Notably, this rate constant increased with increasing initial concentration of Cr (VI) ions and decreasing ABHG dose. The observed phenomena can be attributed to the progressive decrease in the surface area and pore volume of the ABHG adsorbent throughout the separation procedure.

#### Film diffusion model (FDM)

The film diffusion model (FDM) describes the process by which adsorbate molecules migrate through the liquid film surrounding the adsorbent particle. The equation representing FDM is denoted as Eq. (18).

18 $$\mathrm{ln}\left(1-F\right)={K}_{FD}(t)$$

where *K*_FD_ represents the external film mass transfer coefficient, and *F* is the ratio of *q*_t_ to *q*_e_.

The determination of the constant *K*_FD_ can be achieved by computing the slope and intercept of the logarithm of the function (1 − *F*) concerning the plot (Figs. 9e and 10e)^103^. The PSOM was identified as the most appropriate kinetic model for the electrochemical removal of MB dye and Cr (VI) ions onto ABHG. The result above was derived from the observation that the straight lines did not intersect at the sources. These findings suggest that film diffusion is not the rate-limiting step in the overall adsorption kinetics. PSOM rate data yielded the highest determination coefficient (*R*^2^ = 1). The early phase of the process is believed to involve electrostatic attraction between the negatively charged active sites on the self-doped activated carbon and hydrogen ions in solution. This hypothesis is supported by the PSOM, LIM, and TIM adsorption isotherm models. The chemical above consisted of several nitrogen atoms that exhibited unshared electron pairs. The surface of the ABGH, which had undergone positive self-doping, exhibited successful absorption of both MB dye and Cr (VI) ions, forming a distinct adsorption layer.

### Comparison with the result reported in the literature

The literature review conducted a comparative analysis of several adsorbents for their efficacy in removing MB dye and Cr (VI) ions, specifically focusing on the ABHG adsorbent. Table 3 presents a comparative examination of the adsorption capacities of the adsorbents employed in this study, expressed in mg/g, alongside previous findings reported in the current literature. The results in Table 3 indicate that MB dye and Cr(VI) ions were more effectively removed by the ABHG adsorbent than in previous studies using aminobiochar or hydrogel.

**Table 3:** An analysis comparing the maximum capacities of several adsorbents for pollutant removal.

Adsorbent	Pollutant	*Q*_*m*_ (mg/g)	References
Co-pyrolysis of lignin and sewage sludge (SS)	MB dye	154.06	^20^
Sawdust biochar-O_3_-TETA (SDBT)	MB dye	568.16	^27^
Sugar beet pulp (SBP)	MB dye	714.29	^105^
Metal − organic framework (MOF) ZJU-48	MB dye	582.44	^106^
Gigantochloa Bamboo-Derived Biochar	MB dye	86.63	^107^
Cashew nut shell-derived activated carbon	MB dye	456.00	^108^
Amino Acid-Based Hydrophobic Cryogel, Poly(HEMA-MAPA)	MB dye	1304.60	^109^
Wheat straw and E.adenophorum	Cr (VI) ions	88.57	^110^
Magnetite nanoparticles	Cr (VI) ions	34.9	^111^
Fe-modified activated carbon from aquatic plant residue (THAC-Fe)	Cr (VI) ions	11.83	^112^
Rice-husk derived magnetic sorbent (RHC-Mag-2)	Cr (VI) ions	157.7	^113^
*Pterocladia capillacea* red algae its activated carbon	Cr (VI) ions	66.67	^114^
Active carbon derived from Lantana Camara Plant	Cr (VI) ions	26.25	^115^
Chitosan-malonic acid film	Cr (VI) ions	687.05	^116^
Chitin @metakaolin composite	Cr (VI) ions	278.88	^117^
Glutamic acid crosslinked chitosan membrane	Cr (VI) ions	410.70	^118^
Chitosan biopolymer	Cr (VI) ions	340.10	^119^
Amino-functionalized magnetic biochar	Cr (VI) ions	142.86	^120^
Amino-modified upcycled biochar	Cr (VI) ions	46.50	^121^
A fluorescent magnetic chitosan-based hydrogel (FMCH) incorporating Amino-Functionalized Fe_3_O4 nanoparticles (AF-Fe_3_O_4_ NPs) and cellulose nanofibers (CNFs) modified with carbon dots (CDs)	Cr (VI) ions	212.1	^122^
ABHG	MB dye	476.19	This Study
ABHG	Cr (VI) ions	1250.00	This Study

### RSM study

The chosen model was subjected to ANOVA to assess its relevance and identify the factors influencing the elimination percentage^123,124^. The ANOVA findings are presented in Tables S7 and S8. The significance of the factors under investigation and their interactions with the selected answer is demonstrated by the *F*-values. The F-values demonstrate the significance of the factors under investigation and their interactions with the selected answer. The initial concentrations of Cr (VI) and MB dye had the greatest impact on elimination %, as indicated by the data in Tables S9 and S10. Furthermore, terms with *p*-values below 0.05 are deemed significant. The model’s p-value, which is below 0.0001, signifies its statistical significance. Moreover, for Cr (VI), the model’s robustness is further evidenced by the disparity between the adjusted *R*^2^ (0.9764) and the projected *R*^2^ (0.9409), which is below 0.2. (Eqs. 19–22).

The subsequent formulae (19, 20) for the percentage removal of Cr (VI) were derived from the results obtained:

19 $$\begin{aligned} {\text{Removal }}\% {\text{for coded factors}}& = {46}.{88} + { 12}.{\mathrm{86A}} - { 2}.{\mathrm{38B}} + { 21}.{\mathrm{59C}} \hfill \\& \quad + { 8}.{\mathrm{72AB}}{-}{ 4}.{\mathrm{88AC}}{-}{ 9}.{\mathrm{85BC}} \hfill \\ & \quad + { 1}.{\mathrm{15A}}^{{2}} {-}{ 4}.{\mathrm{21B}}^{{2}} + { 5}.0{\mathrm{9C}}^{{2}} \hfill \\ \end{aligned}$$

20 $$\begin{aligned} {\text{Removal }}\% {\text{for actual factors}} &= \, - {7}.{77} + { 7}.{4}0{\mathrm{Dose}} + \, 0.{2}0{\mathrm{Conc}}. \, \hfill \\ \quad & + \, 0.{\mathrm{41Time}} + \, 0.{\text{47 Dose}} \times {\mathrm{Conc}} \hfill \\& - \, 0.{\text{17 Dose }} \times {\mathrm{Time}}. - \, 0.00{\text{3 Conc}}. \, \times {\mathrm{Time}} \hfill \\ \quad & + { 8}.{\text{19 Dose}}^{{2}} {-} \, 0.00{\text{2 Conc}}.^{{2}} - \, 0.00{\text{1 Time}}^{{2}} \hfill \\ \end{aligned}$$

On the other hand, the model’s strength for MB dye is further demonstrated by the difference between the adjusted *R*^2^ (0.9833) and predicted *R*^2^ (0.9663) being less than 0.2. Based on the results obtained, the following Eqs. (21, 22) for MB dye removal % were obtained:

21 $$\begin{aligned} {\text{Removal }}\% {\text{for coded factors}} & = {86}.{1}0 + { 1}.{\mathrm{59A}} - { 2}.{\text{64B }} + { 7}.{\mathrm{65C}} \hfill \\&\quad - \, 0.{\text{21AB }} + 0.0{\text{43AC }} + \, 0.{\mathrm{82BC}} \hfill \\&\quad - \, 0.{6}0{\mathrm{A}}^{{2}} + { 1}.{\mathrm{13B}}^{{2}} - { 1}.{\mathrm{15C}}^{{2}} \hfill \\ \end{aligned}$$

22 $$\begin{aligned} {\text{Removal }}\% {\text{for actual factors}} & = { 77}.{8}0 + { 5}.{\mathrm{17Dose}} - \, 0.{\mathrm{19Conc}}. \, \hfill \\ &\quad + \, 0.{\mathrm{25Time}} - \, 0.00{\text{7Dose }} \times {\mathrm{Conc}} + \, 0.00{\text{2 Dose }} \hfill \\ & \quad \times {\mathrm{Time}}. \, - \, 0.00{\mathrm{1Conc}}. \, \times {\text{Time }}{-}{ 1}.0{\mathrm{7Dose}}^{{2}} \hfill \\ & \quad+ \, 0.00{\mathrm{1Conc}}.^{{2}} - \, 0.00{\mathrm{1Time}}^{{2}} \hfill \\ \end{aligned}$$

Figure 4S shows how the elimination % of Cr (VI) ions is affected by the combined impacts of reaction time, adsorbent dose, and starting Cr (VI) concentration. To achieve the highest removal %, low Cr (VI) concentrations, high ABHG adsorbent doses, and prolonged response times are optimal (Table S9).

Figure 5S shows how the clearance % of MB dye is affected by the combined impacts of reaction time, adsorbent dose, and starting MB dye concentration. The optimal removal % can be achieved with low MB dye concentrations, high ABHG adsorbent doses, and delayed response times (Table S10).

As seen in Figs. S6 and S7, optimal working conditions were numerically determined to achieve the highest percentage of Cr (VI) and MB dye removal.

### ANN modeling for removal of MB dye and Cr(VI) ions

**Fig. 11:**
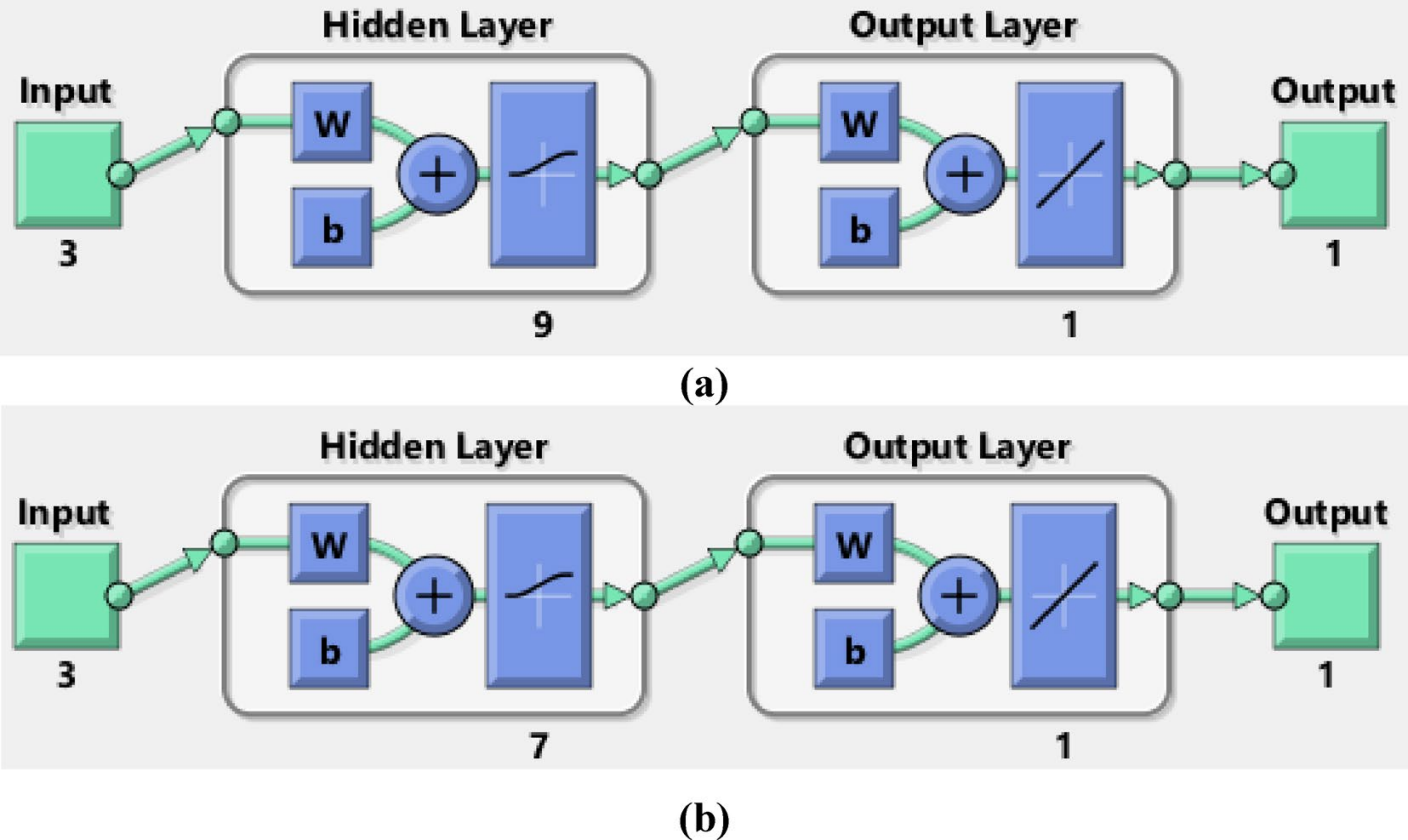
(**a**) ANN architecture for removing MB dye, (**b**) ANN architecture for removing Cr (VI) ions.

**Fig. 12:**
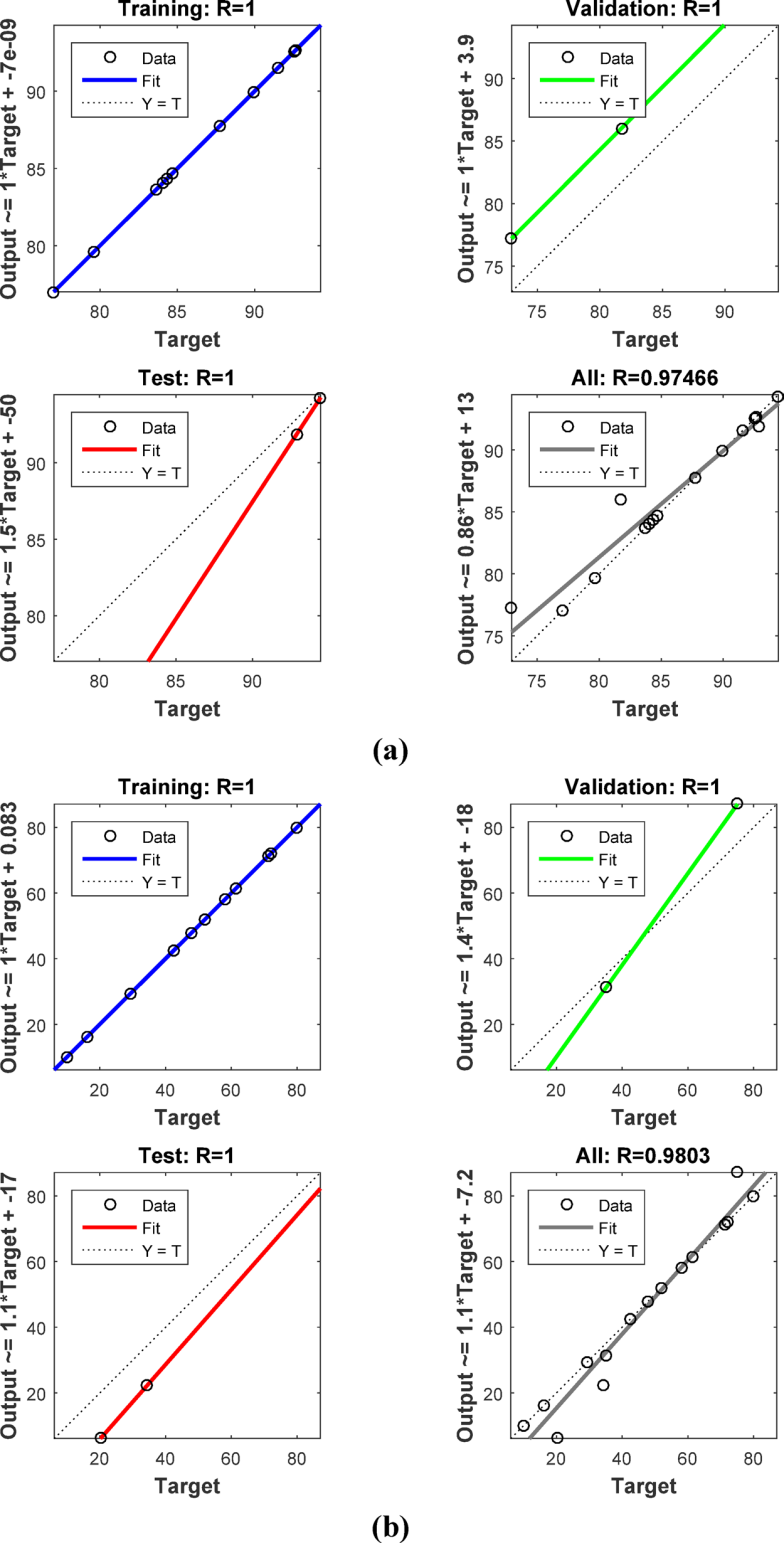
Training, validation, testing, and overall datasets for the LM algorithm of (**a**) MB dye, (**b**) Cr(VI) ions.

**Fig. 13:**
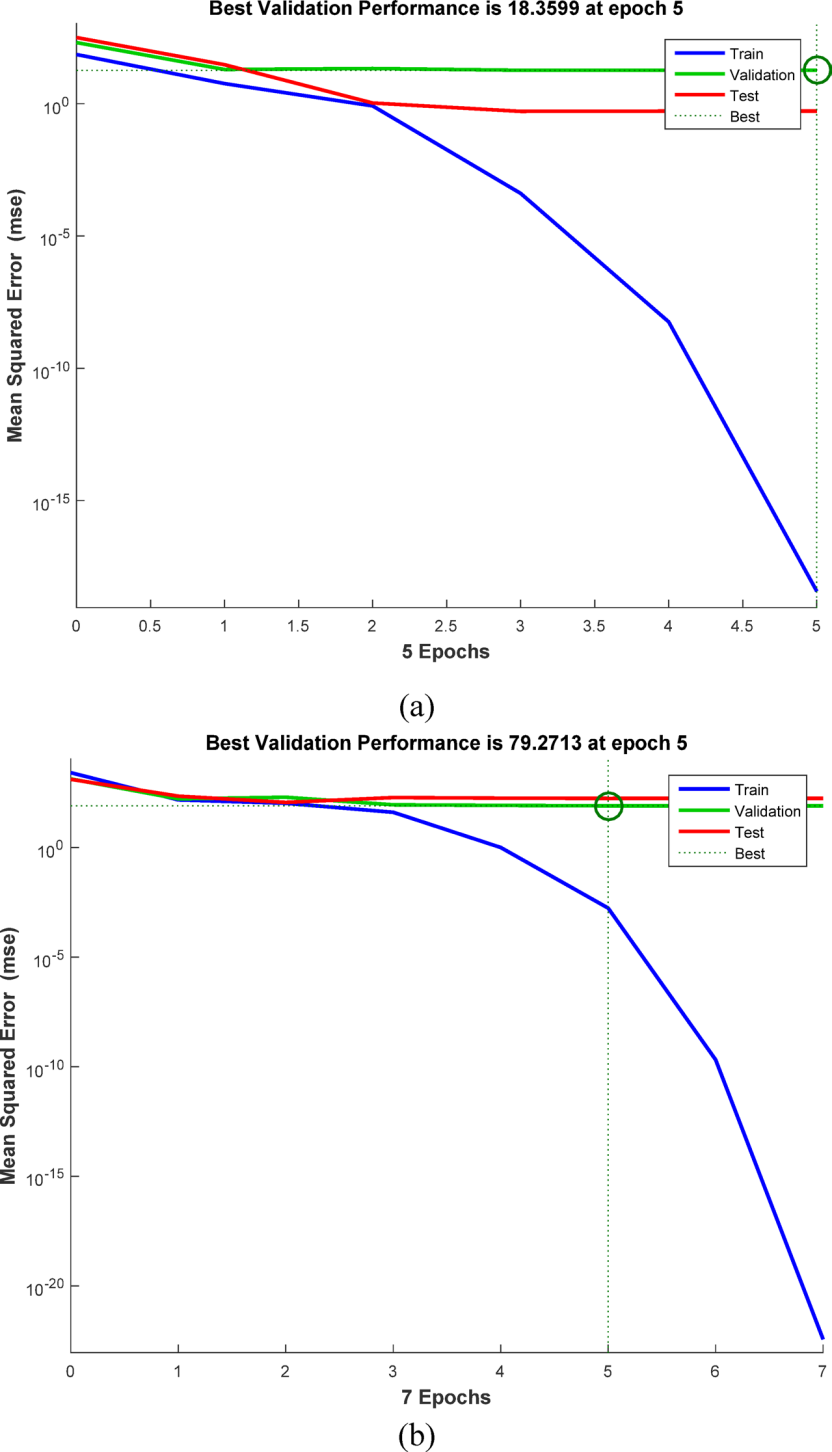
LM algorithm performance, (**a**) MB dye removal, (**b**) Cr (VI) ions removal.

The sample data is composed of the training (70%), testing (15%), and validation (15%). The Levenberg–Marquardt (LM) method trained the ANN model on the sample data. The optimal ANN model for removing MB dye by ABHG is shown in Fig. 11a. The optimal ANN model that showed the removal of MB dye by ABHG was a 3–9–1 architecture (3 neurons in the input layer, 9 neurons in the hidden layer, and 1 neuron in the output layer). The regression plots in Fig. 12a showed that *R*^2^ for training, validation, and testing were 1. *R*^2^ overall was 0.97466. The error value (MSE) was 3.74 × 10^−17^. Log-sigmoid (log-sig) and purelin are the activation functions for the hidden and output layers, respectively. The adsorbent dosage of ABHG (g/L), time (min), and initial concentrations of MB were the 3 independent variables for the best-fit ANN for the adsorption of MB dye, while the removal % of MB dye was the output variable. The best ANN validation performance was observed after 5 epochs and is shown in Fig. 13a^125^.

The training (70%), testing (15%), and validation (15%) are parts of the ANN model’s sample data. Levenberg–Marquardt (LM) was used to train the ANN model on the sample data. The optimal ANN architecture for the elimination of Cr (VI) by ABHG consists of one hidden layer with 7 hidden neurons, 3 inputs (the adsorbent dosage of ABHG (g/L), time (min), and initial concentrations of Cr (VI)), and 1 output (the removal % of Cr (VI)). Figure 11b illustrates this architecture. Figure 12b regression plots show that *R*^2^ for training, validation, and testing was 1, while *R*^2^ overall was 0.9803 (Tables S9 and S10). The 2.31 × 10^−23^ was the error value (MSE). Log-sigmoid (log-sig) was the activation function for the hidden layer. The purelin was the activation function for the output layer. The MSE vs the epoch number is illustrated in Fig. 13b. The MSE vs. epoch number showed perfect performance at 5 epochs for the adsorption process model^126^.

#### Regeneration of ABHG hydrogel adsorbent

**Fig. 14:**
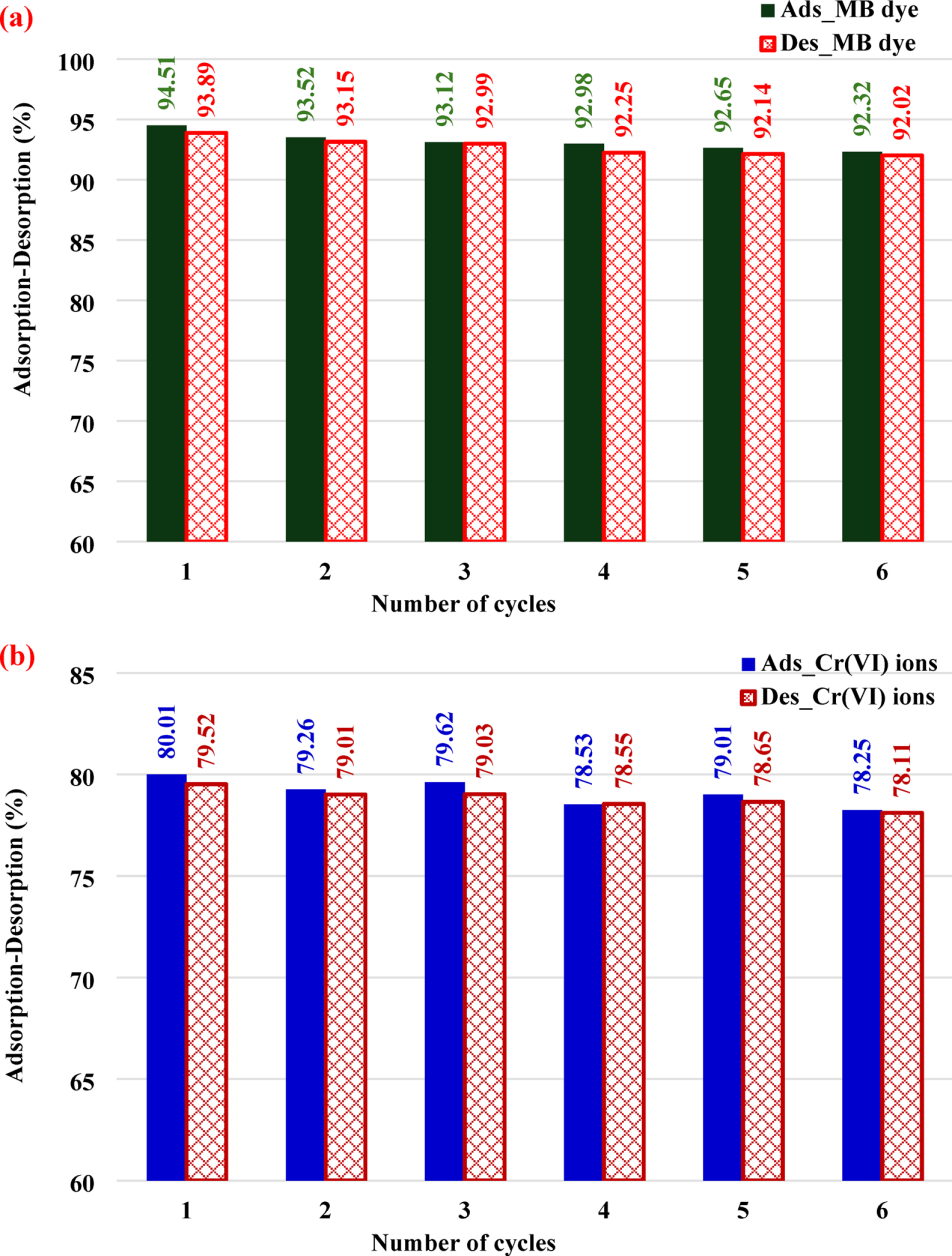
Regeneration of ABHG (1.0 g L^−1^) for adsorption–desorption of (**a**) MB dye (25 mg L^−1^ at pH 7.2), (**b**) Cr (VI) ions (25 mg L^−1^ at pH = 1.04) during 6 cycles.

Desorption studies of the MB dye and Cr (VI) ions from the ABGH adsorbent were conducted using 0.1 M NaOH (100 mL for 30 min) and 0.1 M HCl (100 mL for 30 min) as elution medium and regeneration in order to assess the reusability of ABHG as an adsorbent. In this case, the desorption % decreased somewhat as the number of regeneration cycles increased (Fig. 14). For the two contaminants, the regenerated ABGH was employed in six consecutive adsorption/desorption cycles. Over the six cycles, the amount of adsorption–desorption provided remained mostly consistent. It suggests that it may be used for the adsorption of MB dye and Cr (VI) ions (Fig. 14). More detailed information on the management of solid waste generated after adsorption can be found in the following articles^127–129^.

#### Mechanism of MB dye and Cr(VI) ions adsorption by ABHG

The aminobiochar hydrogel (ABHG) exhibits distinct adsorption mechanisms for MB dye and Cr (VI) ions due to their differing chemical properties and the functional groups present on the ABHG surface. The ABHG, derived from orange peels, possesses a high surface area and diverse functional groups, including nitrogen and oxygen-containing groups such as amine, hydroxyl, and carboxyl groups, which enhance its active site availability and affinity toward both cationic and anionic contaminants^42^.

MB dye is a synthetic cationic dye. The adsorption of MB dye onto ABHG is primarily attributed to chemical adsorption^56^. This is supported by the DRIM analysis, which indicated that the binding energy for MB dye removal exceeded 16 kJ/mol, characteristic of chemical adsorption^56^. Under elevated pH conditions, the acidic sites on the ABHG surface deprotonate, acquiring a negative charge that strongly attracts the cationic MB dye via electrostatic interactions^29^. The PSOM was identified as the most appropriate kinetic model for MB dye removal, suggesting that chemisorption is the rate-limiting step involving electron exchange or sharing between the adsorbent and adsorbate.

Cr (VI) ions are toxic heavy metals that typically exist as anionic forms (HCRO_4_-, Cr_2_O_7_^2-^, and CrO_4_^2−^) in aqueous solutions, with their stability largely dependent on pH^36,52^. The adsorption of Cr (VI) ions onto ABHG is primarily a physical adsorption process^56^. The DRIM showed that the binding energy for Cr (VI) removal was below 8 kJ/mol, indicating physical adsorption^56^. Removal efficiency for Cr(VI) ions was significantly higher at lower pH levels. This is due to the increased concentration of H^+^ ions, which neutralize the negative charge on the adsorbent surface and enhance the diffusion of chromate ions through stronger electrostatic attraction^2,36,52^. The PFOM was found to be suitable for representing the adsorption process of Cr (VI) ions, suggesting that the adsorption rate is proportional to the number of available adsorption sites. The mechanism proposed for the adsorption of the MB dye and Cr (VI) ions by ABHG is explained in Fig. 8S.

## Conclusion

This study demonstrates a sustainable and efficient route to produce functionalized aminobiochar hydrogel (ABHG) from orange peel waste using rapid microwave-assisted H_2_SO_4_ activation followed by oxidation and amination. The proposed synthesis method significantly reduced processing time while yielding a highly porous, nitrogen- and oxygen-enriched carbon matrix. These surface functionalities, combined with the hydrogel network, enhanced adsorption affinity toward both cationic and anionic pollutants.

The adsorption behavior revealed distinct removal mechanisms for each contaminant: methylene blue uptake was governed primarily by chemisorption and electrostatic attraction, while Cr (VI) removal involved reduction–complexation pathways facilitated by surface amine and oxygen groups. Kinetic and isotherm modeling confirmed monolayer adsorption with strong interaction forces, demonstrating the high efficiency and selectivity of ABHG. The maximum adsorption capacity (*Q*_*m*_) for methylene blue was determined to be 476.19 mg/g at an ABHG dosage of 0.5 g/L, whereas a *Q*_*m*_ value of 1250.00 mg/g was obtained for Cr (VI) ions using the Langmuir model at an ABHG dosage of 0.25 g/L. The integration of RSM and ANN provided robust predictive capabilities and optimized key operational parameters, underscoring the applicability of advanced data-driven tools for adsorption system design.

Overall, the results highlight the potential of ABHG as a low-cost, scalable, and environmentally friendly adsorbent for simultaneous treatment of dye- and heavy-metal-contaminated wastewater. Given its rapid synthesis, strong adsorption performance, and reliance on abundant agricultural waste, the developed material represents a promising candidate for industrial wastewater remediation and circular bioeconomy applications. Future work will focus on column-scale studies, regeneration behavior, and application to real effluent systems to support industrial translation.

## Supplementary Information

Below is the link to the electronic supplementary material.

Supplementary Material 1

## Data Availability

The corresponding author of the research can provide the datasets used in this study for review upon request.
