# Supplementary material for: Isotherm, kinetics, and optimization modeling of Cr(VI) ions and methylene blue dye adsorption from water by an aminobiochar hydrogel
Source: Sci Rep. 2026 May 4;16:14172. doi: 10.1038/s41598-026-49810-7 (PMC13139524; doi:10.1038/s41598-026-49810-7)
Supplement: Supplementary file 1 — Supplementary Material 1 [file 41598_2026_49810_MOESM1_ESM.docx]

**Supplementary materials**

**Isotherm, kinetics, and optimization modeling of Cr(VI) ions and methylene blue dye adsorption from water by an aminobiochar hydrogel**

Omnia Fakih Mousa^1^, Murat Yılmaz^2^, Mohamed A. El-Nemr^3,4^, Mohamed A. Hassaan^5^, Amel M. Ismail^1^, Ahmed El Nemr^5,*^

^1^Department of Chemistry, Faculty of Science, Alexandria University, Alexandria, Egypt

^2^Osmaniye Korkut Ata University, Bahçe Vocational School, Department of Chemistry and Chemical Processing Technologies, Osmaniye, 80000, Türkiye

^3^Department of Chemical Engineering, Faculty of Engineering, Minia University, Minia 61519, Egypt

^4^The Higher Canal Institute of Engineering and Technology, Al Salam 1 - Abu Bakr Al Siddiq Street, Suez, Egypt

^5^Environment Division, National Institute of Oceanography and Fisheries (NIOF), Kayet Bey, Elanfoushy, Alexandria, Egypt

E-mail: [fakihomnia@gmail.com](mailto:fakihomnia@gmail.com) (O.F. Mousa); [muratyilmaz@osmaniye.edu.tr](mailto:muratyilmaz@osmaniye.edu.tr) (M. Yılmaz); [mohamedelnemr1992@yahoo.com](mailto:mohamedelnemr1992@yahoo.com) (M.A. El-Nemr); [mhss95@mail.com](mailto:mhss95@mail.com) (M.A. Hassaan); [amelmostafa@yahoo.com](mailto:amelmostafa@yahoo.com) (A.M. Ismail)

* Corresponding Author: E-mail: [ahmedmoustafaelnemr@yahoo.com](mailto:ahmedmoustafaelnemr@yahoo.com); [ahmed.m.elnemr@gmail.com](mailto:ahmed.m.elnemr@gmail.com) (A. El Nemr)

**Figure S1**. Preparation Scheme of the ABHG adsorbent.

| 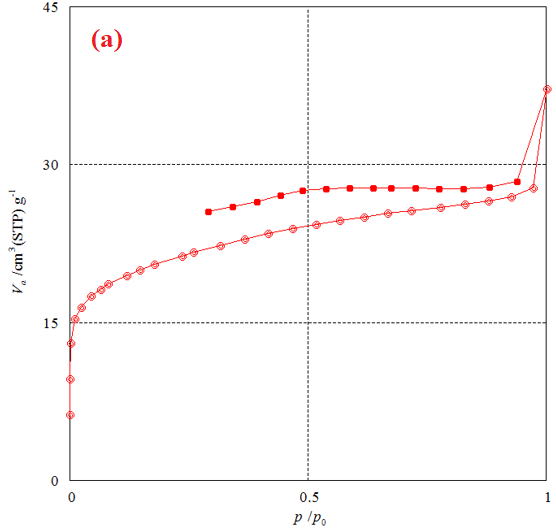 | 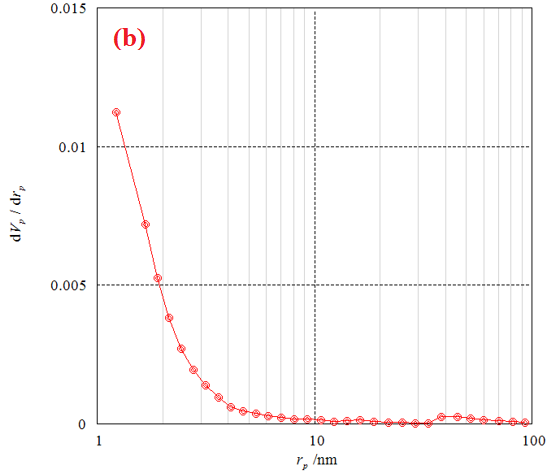 |
| --- | --- |
| 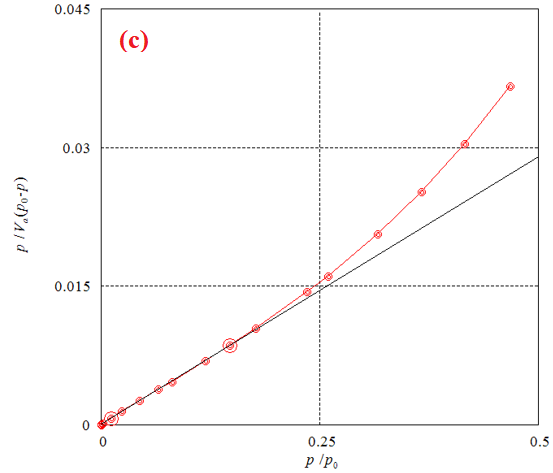 | 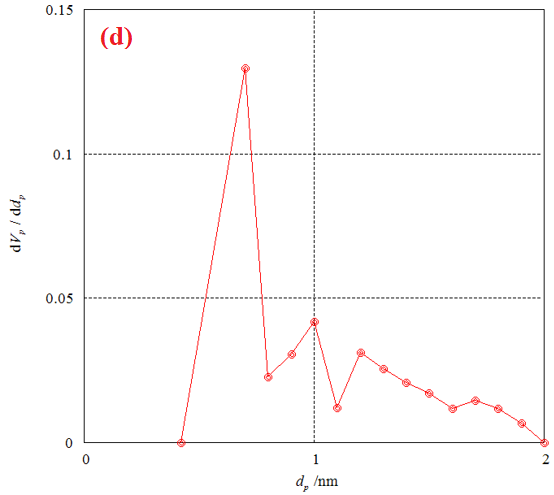 |

**Figure S2**. Graph of N_2_ adsorption-desorption (a), BJH-plot (b), BET-plot (c), MP plot of ABHG.

**
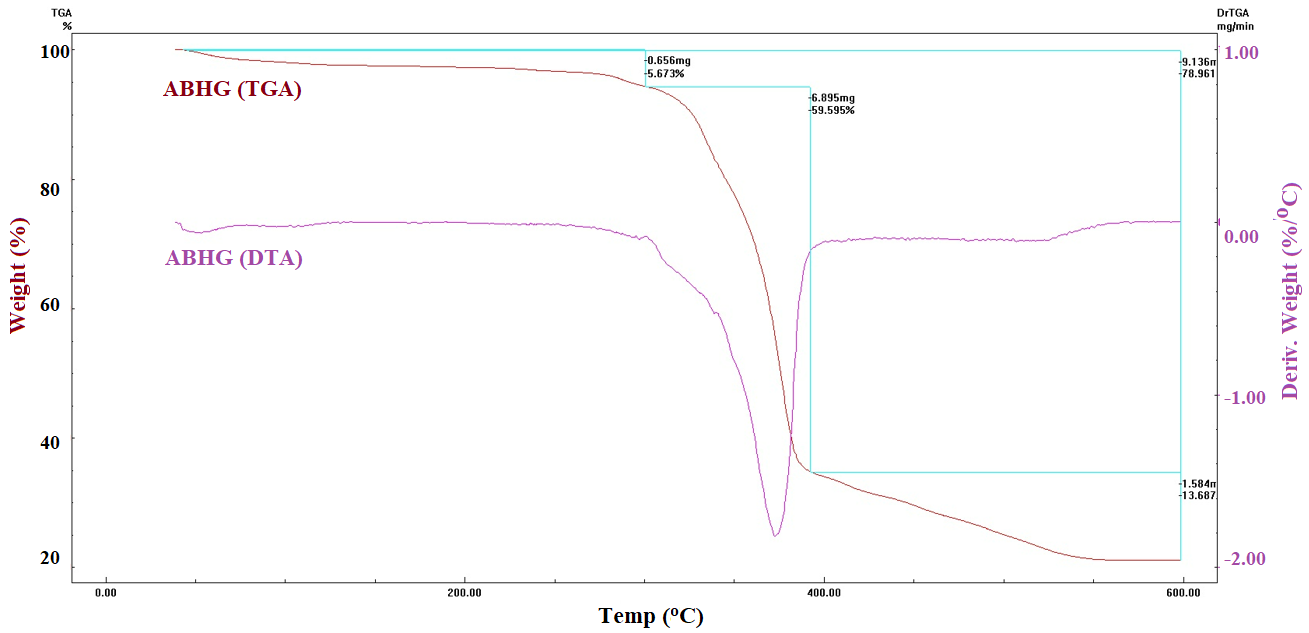
**

**Figure S3.** Diagram of TGA and TDA of ABHG.

**Table S1**. Adsorption isotherm analysis of MB dye adsorption by ABHG at 25 °C.

| **Model** | **Parameters** | **ABHG (g/L)** | | | |
| --- | --- | --- | --- | --- | --- |
|  |  | **0.5** | **1.0** | **1.5** | **2.0** |
| LIM | *Q_m_* | 476.19 | 217.39 | 175.44 | 120.48 |
|  | *K_L_* x 10^3^ | 60.00 | 77.18 | 64.85 | 83.50 |
|  | *R*^2^ | 0.994 | 0.987 | 0.978 | 0.965 |
| FIM | *1/n* | 0.78 | 0.77 | 0.81 | 0.79 |
|  | *K_F_* | 30.01 | 16.93 | 11.35 | 9.81 |
|  | *R*^2^ | 0.999 | 0.991 | 0.993 | 0.992 |
| TIM | *A_T_* | 0.95 | 1.08 | 1.08 | 1.29 |
|  | *B_T_* | 75.69 | 37.98 | 26.77 | 19.65 |
|  | *b* | 32.7 | 65.2 | 92.5 | 126.1 |
|  | *R*^2^ | 0.975 | 0.991 | 0.987 | 0.983 |
| DRIM | *Q_m_* | 156.80 | 81.11 | 55.21 | 41.92 |
|  | *K* × 10^6^ | 1000 | 900 | 900 | 700 |
|  | *E* (KJ mol^-1^) | 22.36 | 23.57 | 23.57 | 26.73 |
|  | *R*^2^ | 0.913 | 0.935 | 0.934 | 0.942 |
| GIM | *N_b_* | 0.99 | 0.86 | 0.87 | 0.83 |
|  | *K_G_* | 16.53 | 28.11 | 41.87 | 48.08 |
|  | *R^2^* | 1.000 | 0.994 | 0.995 | 0.993 |
| HIM | *1/n* | 0.78 | 0.77 | 0.81 | 0.79 |
|  | *k* | 76.67 | 39.04 | 19.90 | 18.15 |
|  | *R*^2^ | 0.999 | 0.991 | 0.993 | 0.992 |

**Table S2**. Adsorption isotherm analysis of Cr (VI) ions adsorption by ABHG at 25 °C

| **Model** | **Parameters** | **ABHG (g/L)** | | | |
| --- | --- | --- | --- | --- | --- |
|  |  | **0.25** | **0.50** | **0.75** | **1.00** |
| LIM | *Q_m_* | 1250.00 | 500.00 | 769.23 | 312.50 |
|  | *K_L_* x 10^3^ | 5.81 | 7.92 | 4.64 | 13.97 |
|  | *R*^2^ | 0.771 | 0.956 | 0.820 | 0.981 |
| FIM | *1/n* | 0.89 | 0.86 | 0.93 | 0.85 |
|  | *K_F_* | 8.82 | 5.13 | 3.98 | 5.28 |
|  | *R*^2^ | 0.991 | 0.995 | 0.998 | 0.996 |
| TIM | *A_T_* | 0.16 | 0.17 | 0.20 | 0.29 |
|  | *B_T_* | 129.87 | 63.70 | 53.22 | 40.18 |
|  | *b* | 19.1 | 38.9 | 46.6 | 61.7 |
|  | *R*^2^ | 0.973 | 0.975 | 0.964 | 0.974 |
| DRIM | *Q_m_* | 237.98 | 119.75 | 94.05 | 76.59 |
|  | *K* × 10^6^ | 22100 | 19700 | 13200 | 6900 |
|  | *E* (KJ mol^–1^) | 4.76 | 5.04 | 6.15 | 8.51 |
|  | *R*^2^ | 0.881 | 0.884 | 0.876 | 0.888 |
| GIM | *N_b_* | 1.01 | 0.91 | 0.10 | 0.88 |
|  | *K_G_* | 178.94 | 269.09 | 338.53 | 246.66 |
|  | *R*^2^ | 0.993 | 0.997 | 0.998 | 0.997 |
| HIM | *1/n* | 0.89 | 0.86 | 0.93 | 0.85 |
|  | *k* | 11.52 | 6.75 | 4.40 | 7.09 |
|  | *R*^2^ | 0.991 | 0.995 | 0.998 | 0.996 |

**Table S3**. Comparing the estimated and observed *q*_e_ values for various starting MB dye and ABHG concentrations and the PFOM and PSOM adsorption rate constants.

| **Parameter** | | |  | **PFOM** | | |  | **PSOM** | | | |
| --- | --- | --- | --- | --- | --- | --- | --- | --- | --- | --- | --- |
| **ABHG**  **(g/L)** | **MB dye**  **(mg/L)** | ***q*_e_ (exp.)** |  | ***k*_1_ × 10^3^** | ***q*_e_ (calc.)** | ***R*^2^** |  | ***k*_2_ × 10^3^** | ***q*_e_**  **(calc.)** | ***h*** | ***R*^2^** |
| 0.5 | 25 | 46.44 |  | 31.32 | 12.35 | 0.992 |  | 4.41 | 48.54 | 10.38 | 0.999 |
|  | 50 | 91.95 |  | 29.94 | 13.65 | 0.955 |  | 4.27 | 94.34 | 38.02 | 1.000 |
|  | 75 | 136.31 |  | 32.47 | 18.22 | 0.943 |  | 3.48 | 138.89 | 67.11 | 1.000 |
|  | 100 | 179.93 |  | 38.69 | 33.58 | 0.924 |  | 2.13 | 185.19 | 72.99 | 1.000 |
| 1.0 | 25 | 23.39 |  | 19.35 | 4.00 | 0.956 |  | 10.30 | 24.10 | 5.98 | 0.998 |
|  | 50 | 46.57 |  | 32.70 | 8.11 | 0.996 |  | 7.62 | 47.85 | 17.45 | 1.000 |
|  | 75 | 69.11 |  | 58.73 | 18.58 | 0.981 |  | 6.52 | 70.92 | 32.79 | 1.000 |
|  | 100 | 90.59 |  | 44.45 | 12.97 | 0.965 |  | 6.55 | 92.59 | 56.18 | 1.000 |
| 1.5 | 25 | 15.63 |  | 25.33 | 3.65 | 0.953 |  | 12.73 | 16.29 | 3.38 | 0.998 |
|  | 50 | 31.15 |  | 38.92 | 5.07 | 0.994 |  | 14.67 | 31.85 | 14.88 | 1.000 |
|  | 75 | 46.41 |  | 43.76 | 6.80 | 0.989 |  | 12.73 | 47.17 | 28.33 | 1.000 |
|  | 100 | 61.04 |  | 49.74 | 10.10 | 0.984 |  | 9.89 | 62.11 | 38.17 | 1.000 |
| 2.0 | 25 | 11.83 |  | 40.07 | 3.05 | 0.993 |  | 23.53 | 12.25 | 3.53 | 1.000 |
|  | 50 | 23.62 |  | 36.62 | 4.10 | 0.996 |  | 16.84 | 24.21 | 9.87 | 1.000 |
|  | 75 | 34.95 |  | 60.80 | 8.01 | 0.992 |  | 16.03 | 35.71 | 20.45 | 1.000 |
|  | 100 | 46.30 |  | 26.25 | 4.30 | 0.977 |  | 12.93 | 46.95 | 28.49 | 1.000 |

**Table S4**. Comparison of the adsorption rate constants for EM, IPDM, and FDM for different MB dye and ABHG starting concentrations.

| **ABHG**  **(g/L)** | **MB dye**  **(mg/L)** |  | **EM** | | |  | **IPDM** | | |  | **FDM** | | |
| --- | --- | --- | --- | --- | --- | --- | --- | --- | --- | --- | --- | --- | --- |
|  |  |  | ***β*** | ***α*** | ***R*^2^** |  | ***K*_dif_** | ***C*** | ***R*^2^** |  | ***K*_FD_** | ***C*** | ***R*^2^** |
| 0.5 | 25 |  | 0.24 | 3.15x10^3^ | 0.993 |  | 1.33 | 34.10 | 0.993 |  | 0.03 | 1.32 | 0.992 |
|  | 50 |  | 0.19 | 3.38 x10^6^ | 0.975 |  | 1.61 | 77.15 | 0.936 |  | 0.03 | 1.91 | 0.955 |
|  | 75 |  | 0.14 | 3.11 x10^7^ | 0.958 |  | 2.14 | 116.96 | 0.901 |  | 0.03 | 2.01 | 0.943 |
|  | 100 |  | 0.11 | 2.17 x10^7^ | 0.965 |  | 3.02 | 152.24 | 0.973 |  | 0.04 | 1.68 | 0.933 |
| 1.0 | 25 |  | 0.64 | 4.27 x10^4^ | 0.921 |  | 0.51 | 18.33 | 0.972 |  | 0.02 | 1.77 | 0.956 |
|  | 50 |  | 0.35 | 4.57 x10^5^ | 0.997 |  | 0.89 | 38.43 | 0.966 |  | 0.03 | 1.75 | 0.996 |
|  | 75 |  | 0.25 | 1.58 x10^6^ | 0.962 |  | 1.24 | 58.39 | 0.891 |  | 0.06 | 1.31 | 0.981 |
|  | 100 |  | 0.27 | 1.37 x10^9^ | 0.968 |  | 1.17 | 80.20 | 0.921 |  | 0.04 | 1.94 | 0.965 |
| 1.5 | 25 |  | 0.77 | 1.97 x10^3^ | 0.960 |  | 0.43 | 11.57 | 0.992 |  | 0.03 | 1.45 | 0.953 |
|  | 50 |  | 0.61 | 3.15 x10^6^ | 0.980 |  | 0.51 | 26.57 | 0.924 |  | 0.04 | 1.82 | 0.994 |
|  | 75 |  | 0.51 | 4.11 x10^8^ | 0.980 |  | 0.61 | 40.98 | 0.924 |  | 0.04 | 1.92 | 0.989 |
|  | 100 |  | 0.36 | 1.55 x10^8^ | 0.948 |  | 0.84 | 53.69 | 0.871 |  | 0.05 | 1.80 | 0.984 |
| 2.0 | 25 |  | 1.10 | 4.98 x10^3^ | 0.994 |  | 0.29 | 9.26 | 0.963 |  | 0.04 | 1.36 | 0.993 |
|  | 50 |  | 0.73 | 5.36 x10^5^ | 0.991 |  | 0.43 | 19.78 | 0.948 |  | 0.04 | 1.75 | 0.996 |
|  | 75 |  | 0.57 | 1.05 x10^7^ | 0.939 |  | 0.54 | 30.35 | 0.855 |  | 0.06 | 1.47 | 0.992 |
|  | 100 |  | 0.60 | 2.45 x10^10^ | 0.987 |  | 0.53 | 41.38 | 0.972 |  | 0.03 | 2.38 | 0.977 |

**Table S5**. Comparing the estimated and observed *q*_e_ values for various beginning Cr (VI) ion concentrations and ABHG concentrations, together with the PFOM and PSOM adsorption rate constants.

| **Parameter** | | |  | **PFOM** | | |  | **PSOM** | | | |
| --- | --- | --- | --- | --- | --- | --- | --- | --- | --- | --- | --- |
| **ABHG**  **(g/L)** | **Cr (VI)**  **(mg/L)** | ***q*_e_ (exp.)** |  | ***k*_1_ × 10^3^** | ***q_e_* (calc.)** | ***R*^2^** |  | ***k*_2_ × 10^3^** | ***q*_e_**  **(calc.)** | ***h*** | ***R*^2^** |
| 0.25 | 25 | 62.67 |  | 19.35 | 108.14 | 0.980 |  | 0.002 | 384.62 | 0.33 | 0.157 |
|  | 50 | 122.67 |  | 16.81 | 163.23 | 0.999 |  | 0.009 | 344.83 | 1.12 | 0.800 |
|  | 75 | 185.11 |  | 13.13 | 191.65 | 0.999 |  | 0.020 | 333.33 | 2.24 | 0.992 |
|  | 100 | 244.22 |  | 12.90 | 244.29 | 0.997 |  | 0.019 | 416.67 | 3.21 | 0.993 |
|  | 125 | 283.78 |  | 8.98 | 230.25 | 0.979 |  | 0.024 | 416.67 | 4.09 | 0.942 |
| 0.50 | 25 | 32.29 |  | 18.19 | 49.81 | 0.992 |  | 0.000 | 833.33 | 0.21 | 0.010 |
|  | 50 | 63.43 |  | 15.43 | 78.23 | 0.999 |  | 0.022 | 163.93 | 0.60 | 0.795 |
|  | 75 | 93.00 |  | 13.59 | 97.84 | 0.999 |  | 0.043 | 163.93 | 1.16 | 0.997 |
|  | 100 | 122.29 |  | 13.59 | 121.81 | 0.992 |  | 0.043 | 200.00 | 1.73 | 0.994 |
|  | 125 | 144.71 |  | 10.59 | 123.20 | 0.991 |  | 0.046 | 217.39 | 2.17 | 0.970 |
| 0.75 | 25 | 24.00 |  | 17.04 | 35.88 | 0.994 |  | 0.000 | 2000.00 | 0.15 | 0.938 |
|  | 50 | 47.52 |  | 14.97 | 59.76 | 0.995 |  | 0.021 | 140.85 | 0.41 | 0.933 |
|  | 75 | 71.14 |  | 6.22 | 81.79 | 0.994 |  | 0.042 | 135.14 | 0.77 | 0.943 |
|  | 100 | 94.667 |  | 9.21 | 84.78 | 0.989 |  | 0.041 | 163.93 | 1.11 | 0.910 |
|  | 125 | 114.86 |  | 9.21 | 98.22 | 0.991 |  | 0.043 | 185.19 | 1.49 | 0.001 |
| 1.00 | 25 | 20.00 |  | 16.81 | 27.62 | 0.989 |  | 0.024 | 80.65 | 0.16 | 0.440 |
|  | 50 | 39.79 |  | 13.82 | 45.16 | 0.999 |  | 0.047 | 91.74 | 0.40 | 0.917 |
|  | 75 | 58.50 |  | 13.59 | 64.65 | 0.992 |  | 0.046 | 117.65 | 0.64 | 0.986 |
|  | 100 | 77.07 |  | 12.90 | 78.78 | 0.998 |  | 0.050 | 136.99 | 0.94 | 0.989 |
|  | 125 | 93.86 |  | 10.82 | 88.94 | 0.998 |  | 0.044 | 161.29 | 1.15 | 0.962 |

**Table S6**. Comparison of the EM, IPDM, and FDM adsorption rate constants for various initial Cr (VI) ions and ABHG concentrations

| **ABHG**  **(g/L)** | **Cr (VI) (mg/L)** |  | **EM** | | |  | **IPDM** | | |  | **FDM** | | |
| --- | --- | --- | --- | --- | --- | --- | --- | --- | --- | --- | --- | --- | --- |
|  |  |  | ***β*** | ***α*** | ***R*^2^** |  | ***K*_dif_** | ***C*** | ***R*^2^** |  | ***K*_FD_** | ***C*** | **R^2^** |
| 0.25 | 25 |  | 0.03 | 1.26 | 0.980 |  | 7.21 | -31.24 | 0.983 |  | 0.02 | -0.55 | 0.980 |
|  | 50 |  | 0.02 | 2.88 | 0.998 |  | 12.09 | -35.57 | 0.990 |  | 0.02 | -0.29 | 0.999 |
|  | 75 |  | 0.01 | 4.87 | 0.990 |  | 16.03 | -29.82 | 0.999 |  | 0.01 | -0.03 | 0.999 |
|  | 100 |  | 0.01 | 6.77 | 0.990 |  | 20.48 | -30.53 | 0.999 |  | 0.13 | 0.00 | 1.000 |
|  | 125 |  | 0.01 | 8.41 | 0.956 |  | 21.80 | -17.96 | 0.979 |  | 0.01 | 0.21 | 0.979 |
| 0.50 | 25 |  | 0.06 | 0.683 | 0.986 |  | 3.52 | -13.61 | 0.979 |  | 0.02 | -0.43 | 0.992 |
|  | 50 |  | 0.04 | 1.51 | 0.999 |  | 6.10 | -16.82 | 0.991 |  | 0.02 | -0.21 | 0.999 |
|  | 75 |  | 0.03 | 2.50 | 0.992 |  | 8.01 | -13.99 | 0.999 |  | 0.01 | -0.05 | 0.999 |
|  | 100 |  | 0.02 | 3.56 | 0.996 |  | 10.07 | -11.76 | 0.994 |  | 0.01 | 0.00 | 0.999 |
|  | 125 |  | 0.02 | 4.43 | 0.976 |  | 11.18 | -7.71 | 0.989 |  | 0.01 | 0.16 | 0.991 |
| 0.75 | 25 |  | 0.09 | 0.497 | 0.992 |  | 2.62 | -10.40 | 0.990 |  | 0.02 | -0.40 | 0.994 |
|  | 50 |  | 0.05 | 1.07 | 0.992 |  | 4.68 | -14.55 | 0.998 |  | 0.01 | -0.23 | 0.995 |
|  | 75 |  | 0.04 | 1.73 | 0.964 |  | 6.18 | -13.97 | 0.990 |  | 0.01 | 0.09 | 0.994 |
|  | 100 |  | 0.03 | 2.41 | 0.961 |  | 7.94 | -14.92 | 0.984 |  | 0.01 | 0.11 | 0.989 |
|  | 125 |  | 0.02 | 3.13 | 0.957 |  | 9.23 | -12.83 | 0.982 |  | 0.01 | 0.16 | 0.991 |
| 1.00 | 25 |  | 0.11 | 0.447 | 0.983 |  | 2.05 | -6.86 | 0.978 |  | 0.02 | -0.32 | 0.989 |
|  | 50 |  | 0.06 | 0.957 | 0.996 |  | 3.72 | -9.64 | 0.994 |  | 0.01 | -0.13 | 0.999 |
|  | 75 |  | 0.04 | 1.45 | 0.984 |  | 5.28 | -12.27 | 0.998 |  | 0.01 | -0.10 | 0.992 |
|  | 100 |  | 0.03 | 2.04 | 0.989 |  | 6.64 | -12.00 | 0.998 |  | 0.01 | -0.02 | 0.998 |
|  | 125 |  | 0.03 | 2.47 | 0.968 |  | 7.86 | -13.65 | 0.995 |  | 0.01 | 0.05 | 0.998 |

**Table S7**. F and p-values ascertained by D-Optimal design for significant variables in Cr (VI) ion elimination

| **Source** | **Sum of Squares** | **df** | **Mean Square** | **F-value** | **p-value** |  |
| --- | --- | --- | --- | --- | --- | --- |
| **Model** | 9268.24 | 9 | 1029.80 | 88.48 | < 0.0001 | **significant** |
| A-Adsorbent dosage | 1908.78 | 1 | 1908.78 | 164.01 | < 0.0001 |  |
| B-Cr(VI) ion Conc. | 64.68 | 1 | 64.68 | 5.56 | 0.0401 |  |
| C-Time | 4740.81 | 1 | 4740.81 | 407.34 | < 0.0001 |  |
| AB | 674.45 | 1 | 674.45 | 57.95 | < 0.0001 |  |
| AC | 199.96 | 1 | 199.96 | 17.18 | 0.0020 |  |
| BC | 793.23 | 1 | 793.23 | 68.16 | < 0.0001 |  |
| A² | 3.73 | 1 | 3.73 | 0.3205 | 0.5838 |  |
| B² | 55.22 | 1 | 55.22 | 4.74 | 0.0544 |  |
| C² | 90.17 | 1 | 90.17 | 7.75 | 0.0193 |  |
| **Residual** | 116.39 | 10 | 11.64 |  |  |  |
| Lack of Fit | 116.39 | 5 | 23.28 |  |  |  |
| Pure Error | 0.0000 | 5 | 0.0000 |  |  |  |
| **Cor Total** | 9384.63 | 19 |  |  |  |  |
| **Std. Dev.** | 3.41 | **R²** | 0.9876 |  |  |  |
| **Mean** | 49.66 | **Adjusted R²** | 0.9764 |  |  |  |
| **C.V. %** | 6.87 | **Predicted R²** | 0.9409 |  |  |  |
| **Adeq Precision** | 29.4967 |  |  |  |  |  |

**Table S8**. F and p-values ascertained by D-Optimal design for significant variables in MB dye elimination

| **Source** | **Sum of Squares** | **df** | **Mean Square** | **F-value** | **p-value** |  |
| --- | --- | --- | --- | --- | --- | --- |
| **Model** | 861.06 | 9 | 95.67 | 124.97 | < 0.0001 | **significant** |
| A-Adsorbent dosage | 27.14 | 1 | 27.14 | 35.45 | 0.0001 |  |
| B-Dye Conc. | 59.84 | 1 | 59.84 | 78.17 | < 0.0001 |  |
| C-Time | 501.45 | 1 | 501.45 | 655.02 | < 0.0001 |  |
| AB | 0.3286 | 1 | 0.3286 | 0.4292 | 0.5272 |  |
| AC | 0.0140 | 1 | 0.0140 | 0.0183 | 0.8952 |  |
| BC | 5.14 | 1 | 5.14 | 6.72 | 0.0268 |  |
| A² | 1.01 | 1 | 1.01 | 1.31 | 0.2782 |  |
| B² | 3.63 | 1 | 3.63 | 4.74 | 0.0545 |  |
| C² | 3.41 | 1 | 3.41 | 4.46 | 0.0609 |  |
| **Residual** | 7.66 | 10 | 0.7655 |  |  |  |
| Lack of Fit | 5.66 | 5 | 1.13 | 2.83 | 0.1393 |  |
| Pure Error | 2.00 | 5 | 0.4000 |  |  |  |
| **Cor Total** | 868.72 | 19 |  |  |  |  |
| **Std. Dev.** | 0.8750 | **R²** | 0.9912 |  |  |  |
| **Mean** | 86.03 | **Adjusted R²** | 0.9833 |  |  |  |
| **C.V. %** | 1.02 | **Predicted R²** | 0.9663 |  |  |  |
| **Adeq Precision** | 33.6095 |  |  |  |  |  |

**Table S9**. Experimental design for Cr (VI) ions removal using ABHG hydrogel adsorbent.

| **Run** | **Factor 1**  **A: Dose**  **(g/L)** | **Factor 2**  **B: Conc.**  **(mg/L)** | **Factor 3 C:Time**  **(min)** | **Experimental**  **Removal %** | **RSM**  **Predicted**  **Removal %** | **ANN**  **Predicted**  **Removal %** |
| --- | --- | --- | --- | --- | --- | --- |
| 1 | 1 | 25 | 90 | 47.85 | 45.23 | 47.85 |
| 2 | 0.5 | 75 | 180 | 71.12 | 71.02 | 71.12 |
| 3 | 1 | 125 | 30 | 61.24 | 61.24 | 61.29 |
| 4 | 1 | 125 | 180 | 75.09 | 74.97 | 75.08 |
| 5 | 1 | 25 | 180 | 80.01 | 81.99 | 80.00 |
| 6 | 0.75 | 25 | 30 | 20.19 | 21.84 | 20.16 |
| 7 | 1 | 125 | 180 | 75.09 | 74.97 | 75.08 |
| 8 | 1 | 75 | 90 | 58.02 | 57.75 | 58.02 |
| 9 | 0.5 | 125 | 90 | 29.43 | 30.74 | 29.46 |
| 10 | 0.25 | 100 | 30 | 16.25 | 12.11 | 16.32 |
| 11 | 0.25 | 25 | 120 | 51.95 | 49.52 | 51.95 |
| 12 | 0.25 | 75 | 120 | 34.37 | 40.66 | 34.24 |
| 13 | 1 | 25 | 180 | 80.01 | 81.99 | 80.00 |
| 14 | 0.25 | 25 | 30 | 9.89 | 10.84 | 9.99 |
| 15 | 1 | 125 | 30 | 61.24 | 61.24 | 61.29 |
| 16 | 0.75 | 50 | 150 | 72.15 | 66.74 | 72.13 |
| 17 | 0.25 | 125 | 180 | 42.31 | 41.55 | 42.29 |
| 18 | 0.75 | 75 | 30 | 35.19 | 36.42 | 35.34 |
| 19 | 0.5 | 125 | 90 | 29.43 | 30.74 | 29.46 |
| 20 | 0.25 | 125 | 180 | 42.31 | 41.55 | 42.29 |

**Table S10**. Experimental design for MB dye removal using ABHG hydrogel adsorbent.

| **Run** | **Factor 1**  **A: Dose**  **(mg)** | **Factor 2**  **B: Conc.**  **(mg/L)** | **Factor 3**  **C: Time**  **(min)** | **Experimental**  **Removal %** | **RSM**  **Predicted**  **Removal %** | **ANN**  **Predicted**  **Removal %** |
| --- | --- | --- | --- | --- | --- | --- |
| 1 | 2 | 25 | 60 | 92.55 | 92.40 | 92.55 |
| 2 | 0.5 | 100 | 90 | 89.96 | 89.90 | 89.96 |
| 3 | 1 | 25 | 15 | 81.72 | 81.24 | 81.03 |
| 4 | 2 | 75 | 15 | 77.02 | 77.16 | 77.02 |
| 5 | 0.5 | 100 | 15 | 72.95 | 73.05 | 72.21 |
| 6 | 0.5 | 25 | 90 | 92.89 | 93.12 | 92.88 |
| 7 | 2 | 100 | 90 | 92.61 | 92.75 | 92.61 |
| 8 | 1.5 | 25 | 30 | 84.67 | 85.89 | 84.67 |
| 9 | 2 | 50 | 30 | 84.07 | 83.30 | 84.07 |
| 10 | 0.5 | 50 | 45 | 82.35 | 83.34 | 84.35 |
| 11 | 1.5 | 100 | 45 | 83.65 | 83.25 | 83.65 |
| 12 | 1.5 | 50 | 60 | 87.75 | 89.03 | 87.75 |
| 13 | 0.5 | 100 | 90 | 89.96 | 89.90 | 89.96 |
| 14 | 0.5 | 25 | 90 | 92.89 | 93.12 | 92.88 |
| 15 | 1 | 25 | 60 | 91.52 | 90.52 | 91.52 |
| 16 | 1 | 75 | 30 | 79.63 | 79.62 | 79.63 |
| 17 | 2 | 100 | 90 | 92.61 | 92.75 | 92.61 |
| 18 | 0.5 | 50 | 45 | 84.35 | 83.34 | 84.35 |
| 19 | 1.5 | 50 | 90 | 94.45 | 93.84 | 94.45 |
| 20 | 0.5 | 100 | 15 | 72.95 | 73.05 | 72.21 |

| 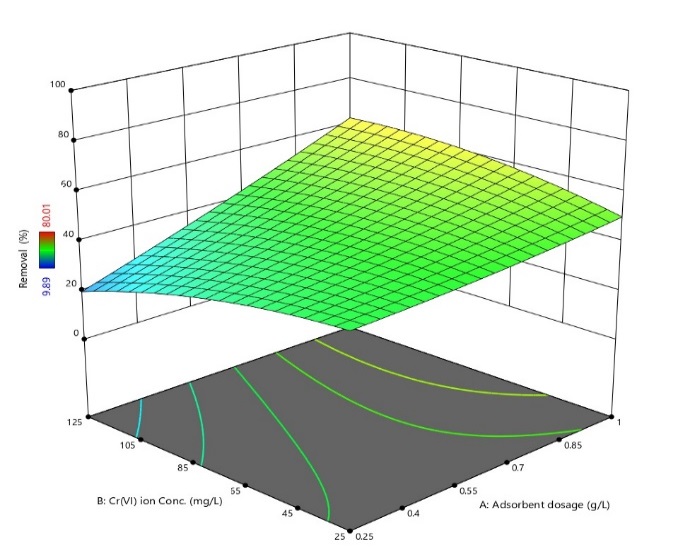  **a** | 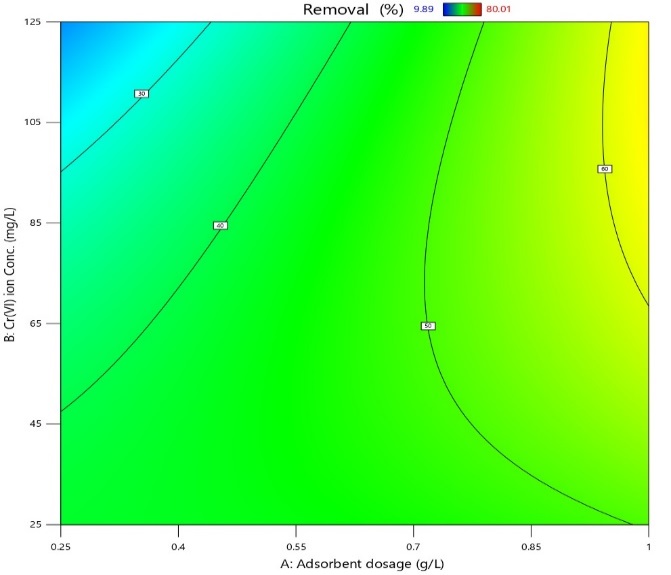  **b** |
| --- | --- |
| 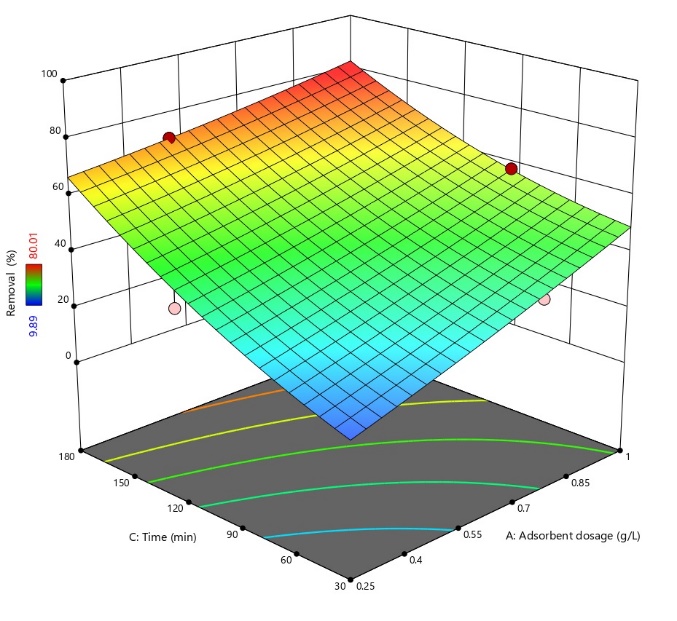  **c** | 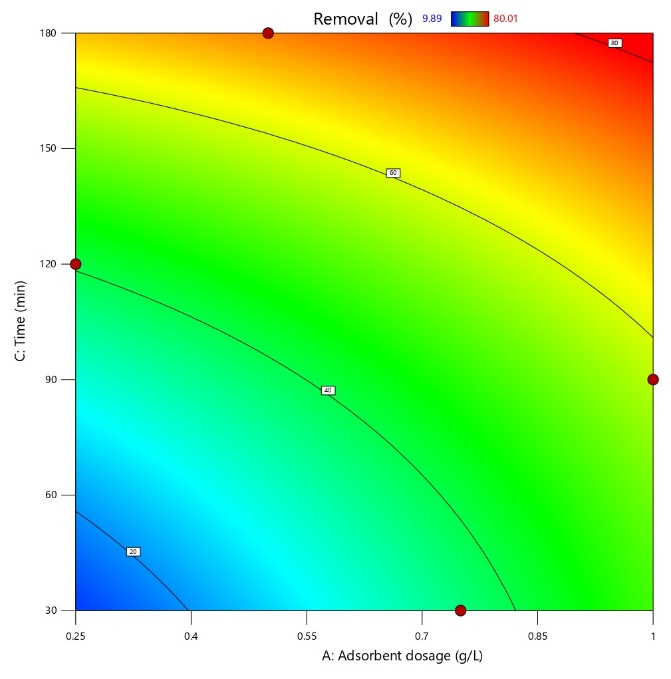  **d** |
| 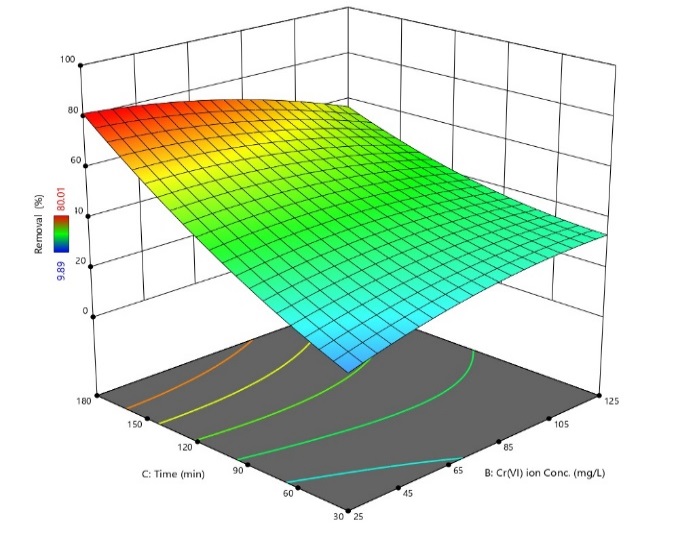  **e** | 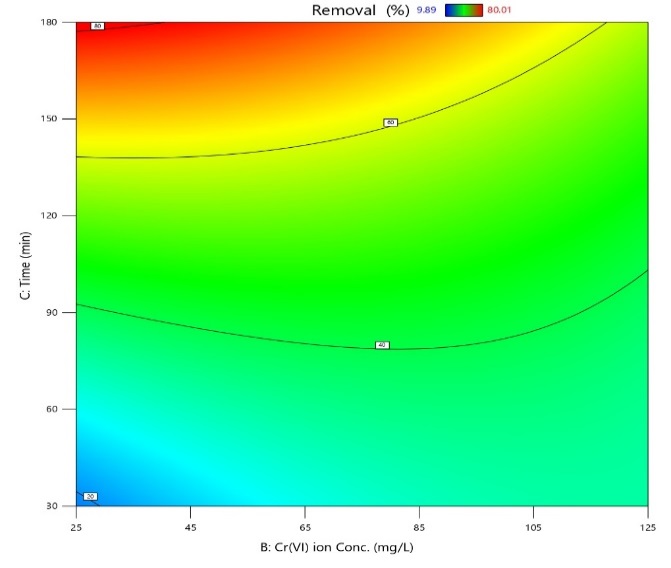  **f** |

**Figure 4S**. Combined effects of independent variables: (a, b) ABHG adsorbent dosage and Cr (VI) initial concentration, (c, d) ABHG Adsorbent dosage and time, and (e, f) Cr (VI) initial concentration and time.

| 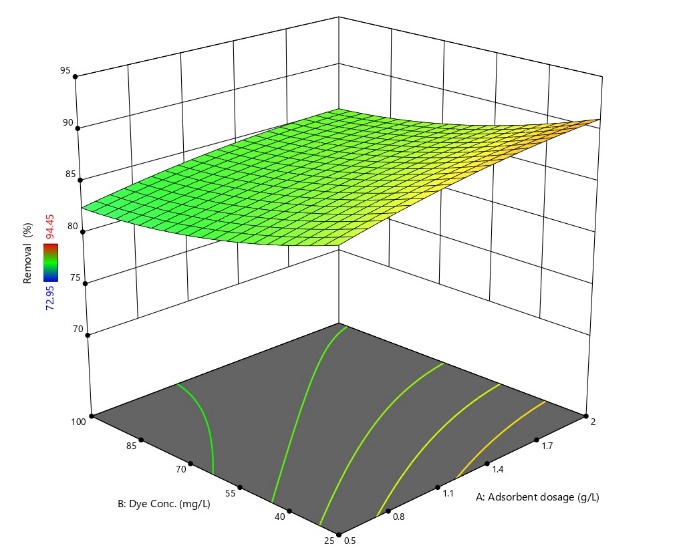  **a** | 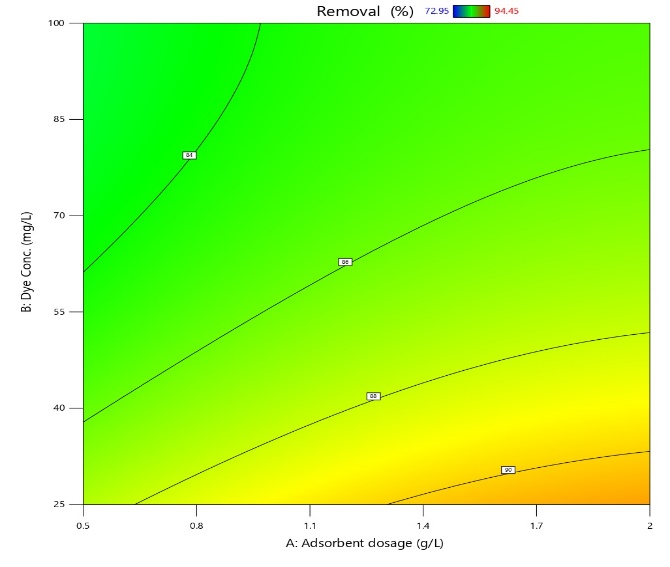  **b** |
| --- | --- |
| 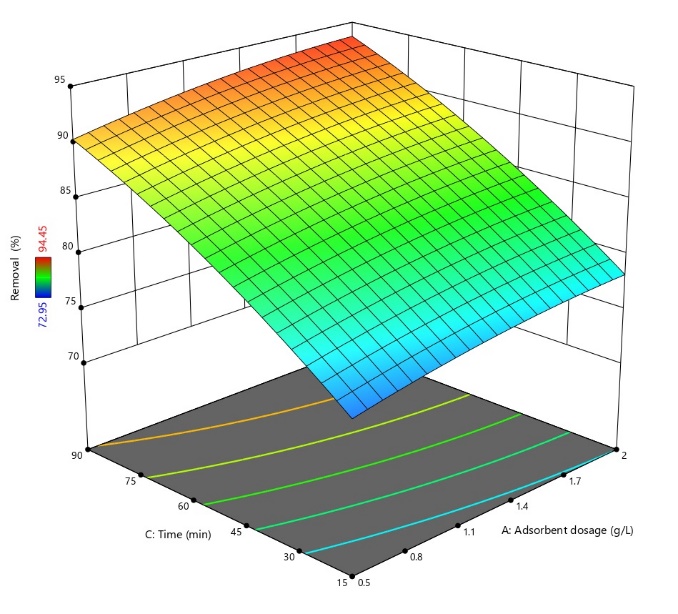  **c** | 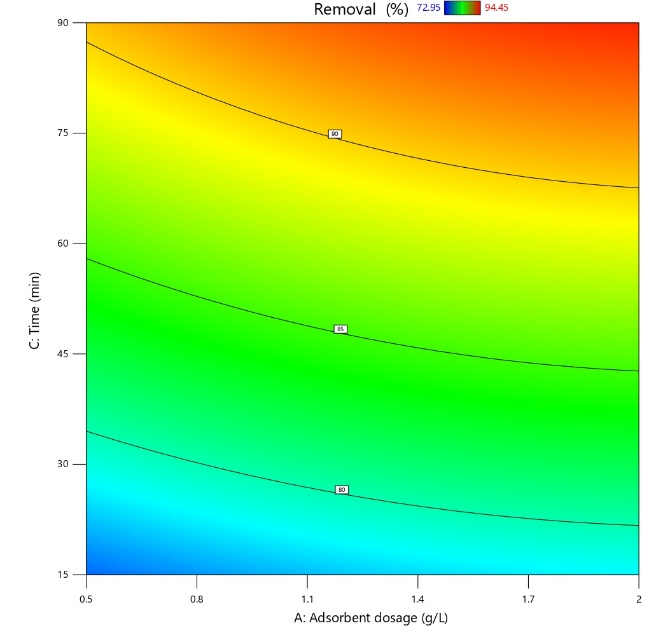  **d** |
| 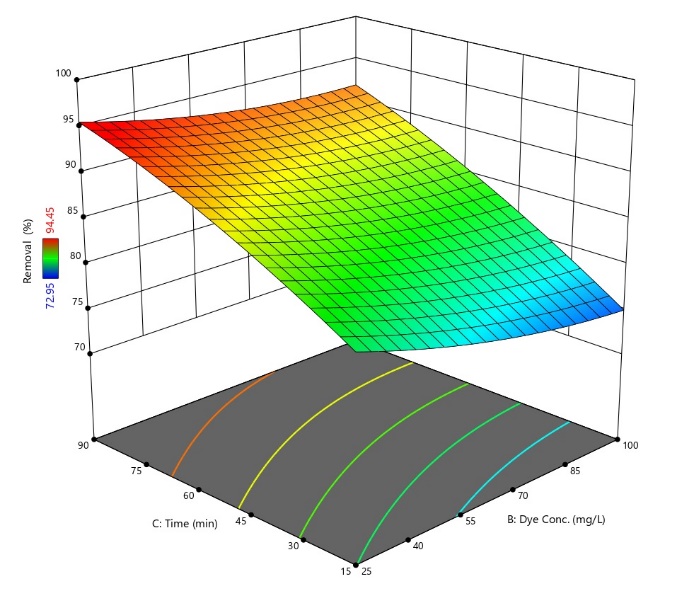  **e** | 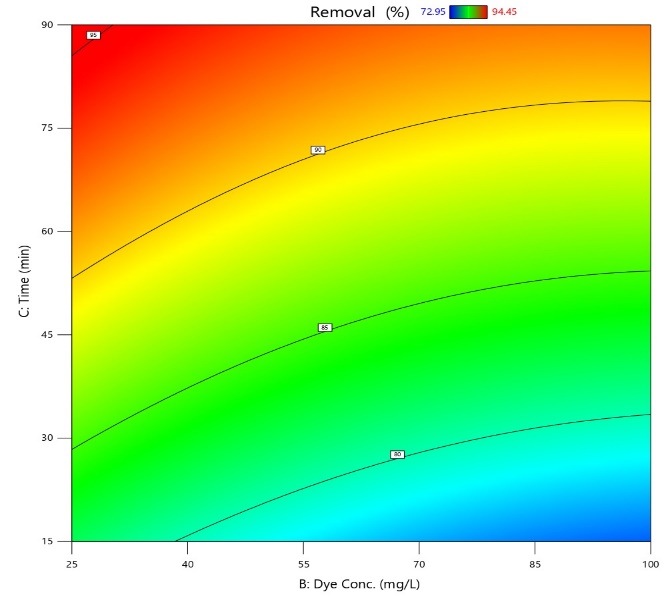  **f** |

**Figure 13**. Combined effects of independent variables: (a, b) ABHG Adsorbent dosage and MB dye initial concentration, (c, d) ABHG Adsorbent dosage and time, and (e, f) MB dye initial concentration and time.


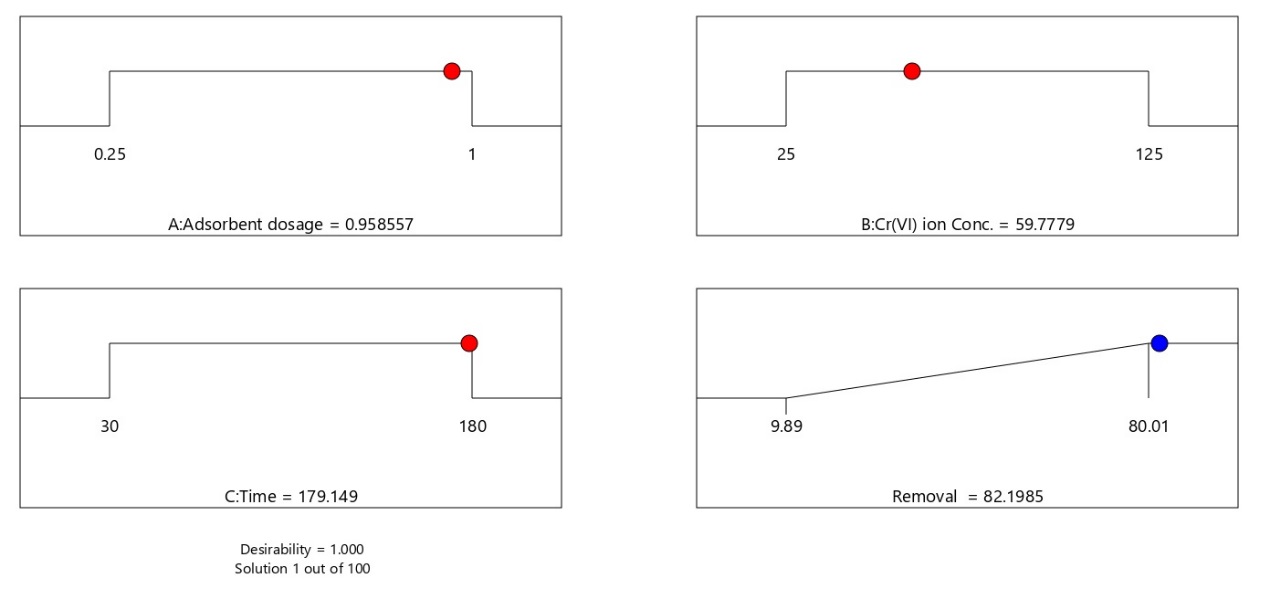


**Figure S6**. Optimization conditions through DOD settings.


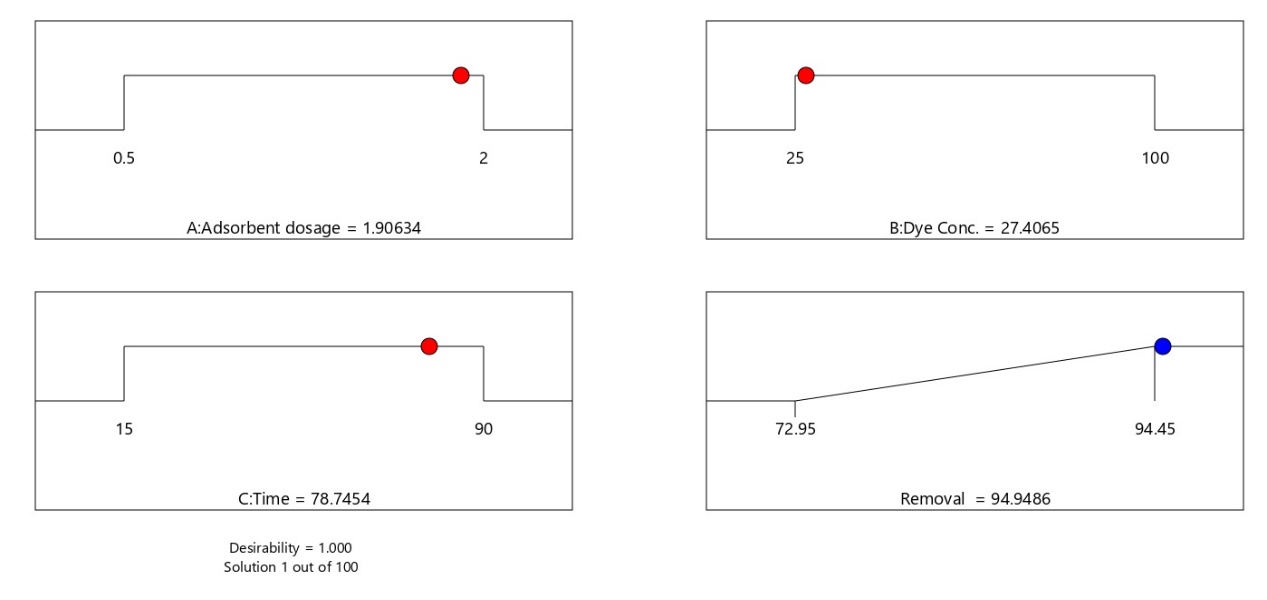


**Figure S7**. Optimization conditions through DOD settings.


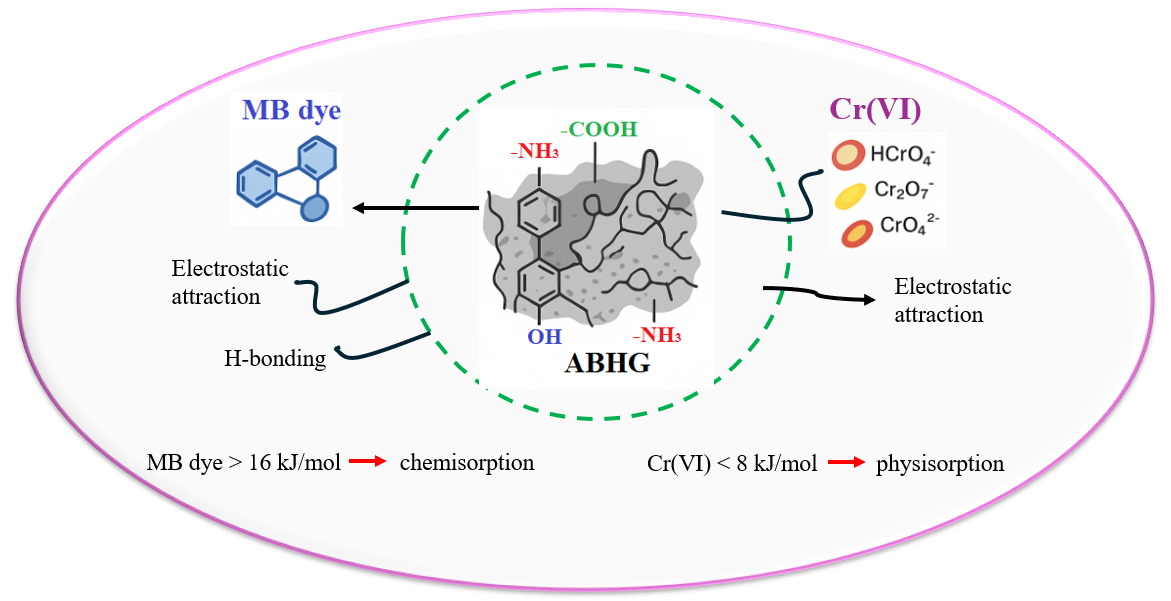


**Figure 8S.** Proposed mechanism for the MB dye and Cr(VI) ions uptake from water using ABHG adsorbent.
